# On Evaluating MHC-II Binding Peptide Prediction Methods

**DOI:** 10.1371/journal.pone.0003268

**Published:** 2008-09-24

**Authors:** Yasser EL-Manzalawy, Drena Dobbs, Vasant Honavar

**Affiliations:** 1 Department of Computer Science, Center for Computational Intelligence, Learning, and Discovery, Iowa State University, Ames, Iowa, United States of America; 2 Department of Genetics, Development and Cell Biology, Bioinformatics and Computational Biology Graduate Program, Center for Computational Intelligence, Learning and Discovery, Iowa State University, Ames, Iowa, United States of America; University of the Western Cape, South Africa

## Abstract

Choice of one method over another for MHC-II binding peptide prediction is typically based on published reports of their estimated performance on standard benchmark datasets. We show that several standard benchmark datasets of *unique peptides* used in such studies contain a substantial number of peptides that share a high degree of sequence identity with one or more other peptide sequences in the same dataset. Thus, in a standard cross-validation setup, the test set and the training set are likely to contain sequences that share a high degree of sequence identity with each other, leading to overly optimistic estimates of performance. Hence, to more rigorously assess the relative performance of different prediction methods, we explore the use of *similarity-reduced* datasets. We introduce three *similarity-reduced* MHC-II benchmark datasets derived from MHCPEP, MHCBN, and IEDB databases. The results of our comparison of the performance of three MHC-II binding peptide prediction methods estimated using datasets of *unique* peptides with that obtained using their *similarity-reduced* counterparts shows that the former can be rather optimistic relative to the performance of the same methods on *similarity-reduced* counterparts of the same datasets. Furthermore, our results demonstrate that conclusions regarding the superiority of one method over another drawn on the basis of performance estimates obtained using commonly used datasets of *unique* peptides are often contradicted by the observed performance of the methods on the *similarity-reduced* versions of the same datasets. These results underscore the importance of using *similarity-reduced* datasets in rigorously comparing the performance of alternative MHC-II peptide prediction methods.

## Introduction

T-cells epitopes are short linear peptides generated by cleavage of antigenic proteins. The identification of T-cell epitopes in protein sequences is important for understanding disease pathogenesis, identifying potential autoantigens, and designing vaccines and immune-based cancer therapies. A major step in identifying potential T-cell epitopes involves identifying the peptides that bind to a target major histocompatibility complex (MHC) molecule. Because of the high cost of experimental identification of such peptides, there is an urgent need for reliable computational methods for predicting MHC binding peptides [1].

There are two major classes of MHC molecules: MHC class I (MHC-I) molecules characterized by short binding peptides, usually consisting of nine residues; and MHC class II (MHC-II) molecules with binding peptides that range from 11 to 30 residues in length, although shorter and longer peptide lengths are not uncommon [2]. The binding groove of MHC-II molecules is open at both ends, allowing peptides longer than 9-mers to bind. However, it has been reported that a 9-mer core region is essential for MHC-II binding [2], [3]. Because the precise location of the 9-mer core region of MHC-II binding peptides is unknown, predicting MHC-II binding peptides tends to be more challenging than predicting MHC-I binding peptides.

Despite the high degree of variability in the length of MHC-II binding peptides, most existing computational methods for predicting MHC-II binding peptides focus on identifying a 9-mer core peptide. Computational approaches available for predicting MHC-II binding peptides from amino acid sequences include: (i) Motif-based methods such as methods that use a position weight matrix (PWM) to model an ungapped multiple sequence alignment of MHC binding peptides [4]–[8], and a statistical approach based on Hidden Markov Models (HMMs) [9], [10]; (ii) Machine learning methods based on Artificial Neural Networks (ANN) [6], [11]–[13] and Support Vector Machines (SVMs) [14]–[17]; (iii) Semi-supervised machine learning methods [18], [19].

The choice of one method over another for MHC-II binding peptide prediction requires reliable assessment of their performance relative to each other. Such assessments usually rely on estimates of their performance on standard benchmark datasets (typically obtained using cross-validation). Several studies [5], [15]–[17], [19] have reported the performance of MHC-II binding peptide prediction methods using datasets of *unique* peptides. Such datasets can in fact contain peptide sequences that share a high degree of sequence similarity with other peptide sequences in the dataset. Hence, several authors [6], [7], [10], [20] have proposed methods for eliminating *redundant* sequences. However, because MHC-II peptides have lengths that vary over a broad range, similarity reduction of MHC-II peptides is not a straightforward task [7]. Consequently, standard cross-validation based estimates of performance obtained using such datasets are likely to be overly optimistic because the test set is likely to contain sequences that share significant sequence similarity with one or more sequences in the training set.

In order to obtain more realistic estimates of performance of MHC-II binding peptide prediction methods, we explored several methods for constructing *similarity-reduced* MHC-II datasets. We constructed *similarity-reduced* MHC-II benchmark datasets, derived from MHCPEP [21], MHCBN [22], and IEDB [23] databases, using several approaches to reduce the degree of pair-wise sequence similarity shared by sequences in the resulting datasets. The similarity reduction procedures were applied separately to binders and non-binders. Details of the similarity reduction methods are provided in the Materials and Methods Section. Specifically, we generated:

- Datasets of unique peptides MHCPEP-UPDS, MHCBN-UPDS, and IEDB-UPDS extracted from MHCPEP, MHCBN, and IEDB, respectively.
- Datasets of *similarity-reduced* peptides, MHCPEP-SRDS1, MHCBN-SRDS1, and IEDB-SRDS1 derived from the corresponding UPDS datasets using a similarity reduction procedure which ensures that no two peptides in the resulting dataset share a 9-mer subsequence.
- Datasets of *similarity-reduced* peptides, MHCPEP-SRDS2, MHCBN-SRDS2, and IEDB-SRDS2, extracted MHCPEP-SRDS1, MHCBN-SRDS1, and IEDB-SRDS1 respectively by filtering the binders and non-binders in SRDS1 such that the sequence identity between any pair of peptides is less than 80%.
- Datasets of *similarity-reduced* peptides, MHCPEP-SRDS3, MHCBN-SRDS3, and IEDB-SRDS3, derived from the corresponding UPDS datasets using the similarity reduction procedure introduced by Raghava and previously used to construct the MHCBench dataset [20].
- Datasets of *weighted* unique peptides, MHCPEP-WUPDS, MHCBN-WUPDS, and IEDB-WUPDS, derived from the corresponding UPDS datasets (where the weight assigned to a peptide is inversely proportional to the number of peptides that are similar to it).

We then used the resulting *similarity-reduced* benchmark datasets to explore the effect of similarity reduction on the performance of different MHC-II binding peptide prediction methods and, more importantly, to rigorously compare the performance of the different prediction methods.

Our experiments focused on two state-of-the-art methods for training MHC-II binding peptide predictors using variable-length MHC-II peptides and a third method that is designed to exploit the sequence similarity between a test peptide sequence and the peptide sequences in the training set (and is hence likely to perform well on *non similarity-reduced* datasets but poorly on the *similarity-reduced* datasets).

Specifically, we compared: (i) An approach [16] that maps each variable-length peptide into a fixed-length feature vector (the so-called composition-transition distribution or CTD) consisting of sequence-derived structural features and physicochemical properties of the input peptide sequence; (ii) An approach [17] that uses a local alignment (LA) kernel that defines the similarity between two variable-length peptides as the average of all possible local alignments between the two peptides; (iii) An approach that uses the k-spectrum kernel [24] with *k* = 5.

Because neither the programs used to calculate secondary structure and solvent accessibility of peptides used for generating the CTD representation [16] nor the precise choices of parameters used for training the LA kernel based classifier [17] were available to us, we used in our experiments, our own implementations of the corresponding methods. Hence, the results of our experiments should not be viewed as providing direct assessment of performance of the exact implementations of the CTD and LA methods developed by the original authors and used in studies reported in [16], [17]. However, it is worth noting that, the broad conclusions of our study are largely independent of the specific machine learning methods or data transformations.

Our results demonstrate that, regardless of the similarity reduction method employed, a substantial drop in performance of classifiers is observed compared to their reported performance on benchmark datasets of *unique* peptide sequences. Our results also demonstrate that conclusions regarding the superiority of one prediction method over another can be misleading when they are based on evaluations using benchmark datasets with a high degree of sequence similarity (e.g., the benchmark dataset of *unique* peptide sequences). These results underscore the importance of using *similarity-reduced* datasets in evaluating and comparing alternative MHC-II peptide prediction methods.

## Results

### Limitations of the *unique* peptides MHC-II data

Tables 1–3 show that MHC-II datasets derived from MHCPEP, MHCBN, and IEDB databases have a large number of highly similar peptides: the number of peptides in the *similarity-reduced* versions in the three benchmark datasets is ≈50% of the original number. In each case, the estimated performance of the prediction methods evaluated on *similarity-reduced* datasets is substantially worse than that estimated using the datasets of unique peptides. This finding is especially significant in light of the fact that MHCPEP and MHCBN datasets have been used for comparing alternative MHC-II peptide prediction methods in most of the published studies [5], [6], [15]–[19], [25].

**Table 1:** Number of binding peptides in MHCPEP benchmark dataset.

Allele	UPDS	SRDS1	SRDS2	SRDS3
HLA-DQ2	113	67	32	39
HLA-DQ4	97	84	79	82
HLA-DQ7	135	73	65	75
HLA-DR1	703	336	242	278
HLA-DR2	315	148	104	134
HLA-DR3	192	81	69	73
HLA-DR4	1085	439	298	353
HLA-DR5	189	92	61	75
HLA-DR7	341	137	87	101
HLA-DR8	125	47	46	54
HLA-DR9	94	41	34	37
HLA-DR11	473	160	100	103
HLA-DR13	121	68	34	36
HLA-DR15	121	48	36	49
HLA-DR17	158	82	40	45
HLA-DR51	115	45	39	55
I-Ab	136	62	51	61
I-Ad	415	168	101	135
I-Ag7	157	62	53	81
I-Ak	254	96	67	85
I-Ed	294	188	68	76
I-Ek	334	204	64	78

UPDS refers to datasets of non-redundant peptides. The last three columns refer to *similarity-reduced* datasets (see text for details).

**Table 2 pone-0003268-t002:** Number of binding/non-binding peptides in MHCBN benchmark dataset.

Allele	UPDS	SRDS1	SRDS2	SRDS3
HLA-DR1	636/180	328/111	223/106	259/130
HLA-DR2	416/168	197/124	153/123	232/149
HLA-DR5	218/173	111/131	80/129	100/154
HLA-DRB10101	531/127	325/88	279/76	390/112
HLA-DRB10301	261/230	137/150	127/145	175/215
HLA-DRB10401	805/201	471/136	404/119	543/174
HLA-DRB10701	292/107	179/68	152/66	213/92
HLA-DRB11101	352/137	213/87	182/87	239/131

UPDS refers to datasets of non-redundant peptides. The last three columns refer to *similarity-reduced* datasets (see text for details).

**Table 3:** Number of binding/non-binding peptides in IEDB benchmark dataset.

Allele	UPDS	SRDS1	SRDS2	SRDS3
HLA-DRB1-0101	1105/432	645/268	623/261	938/365
HLA-DRB1-0301	135/556	78/292	69/276	81/396
HLA-DRB1-0401	317/412	197/262	176/255	215/340
HLA-DRB1-0404	113/132	69/100	62/98	74/109
HLA-DRB1-0405	113/119	74/85	70/84	81/89
HLA-DRB1-0701	228/302	147/203	137/202	173/274
HLA-DRB1-0802	65/120	46/101	46/100	49/108
HLA-DRB1-1101	197/411	122/218	111/212	139/328
HLA-DRB1-1302	152/103	105/81	97/81	110/92
HLA-DRB1-1501	269/283	165/176	142/174	185/260
HLA-DRB4-0101	92/215	64/120	63/119	85/200
HLA-DRB5-0101	215/377	123/201	113/194	147/309

UPDS refers to datasets of non-redundant peptides. The last three columns refer to *similarity-reduced* datasets (see text for details).

For the sake of brevity, we focus discussion here on the results of two representative examples of datasets extracted from the MHCPEP and MHCBN benchmarks and provide the complete set of results in the supplementary materials (Data S1).

As shown in Table 4, for the MHCPEP benchmark, we focus on the results on the data for HLA-DR4, which has the largest number of *unique* binders. On the MHCPEP-UPDS version of the HLA-DR4 dataset, the 5-spectrum kernel outperforms the other two prediction methods and CTD outperforms the LA kernel. We notice a substantial drop in the observed performance of the three prediction methods on the *similarity-reduced* and *weighted* datasets relative to that on their UPDS counterpart.

**Table 4:** Performance of prediction methods on MHCPEP HLA-DR4 *unique*, *similarity-reduced*, and *weighted* datasets using 5-fold cross-validation test.

Dataset	Method	ACC	Sn	Sp	CC	AUC
UPDS	CTD	86.59	73.36	99.82	0.759	0.906
	LA	77.10	71.71	82.49	0.545	0.862
	5-spectrum	90.55	81.29	99.82	0.825	0.917
SRDS1	CTD	69.93	66.06	73.80	0.400	0.723
	LA	68.56	63.78	73.35	0.373	0.751
	5-spectrum	70.96	43.28	98.63	0.503	0.710
SRDS2	CTD	64.77	60.40	69.13	0.296	0.692
	LA	64.43	65.10	63.76	0.289	0.711
	5-spectrum	56.04	33.22	78.86	0.136	0.578
SRDS3	CTD	65.01	61.47	68.56	0.301	0.695
	LA	64.02	62.89	65.16	0.281	0.717
	5-spectrum	68.56	38.81	98.30	0.462	0.679
WUPDS	CTD	85.41	31.98	99.91	0.516	0.730
	LA	79.38	22.50	94.81	0.249	0.723
	5-spectrum	87.14	42.14	99.35	0.580	0.723

In the case of the MHCBN benchmark, we focus on the results on the HLA-DRB1*0301 data (Table 5) because it has been used in a number of recent studies of MHC-II binding peptide prediction methods [16], [17], [25]. Most MHCBN allele-specific datasets are unbalanced, i.e., the numbers of binding peptides in the datasets are larger (typically by a factor of 2 to 4) than the corresponding numbers of non-binding peptides (see Table 2). On such unbalanced datasets, classification accuracy can be misleading in terms of providing a reliable and useful assessment of the performance of the classifier. A classifier that simply returns the label of the majority class as the predicted label for each instance to be classified can achieve a rather high accuracy; However such a classifier is rather useless in reliably identifying members of the minority class. Hence, in the case of unbalanced datasets, the correlation coefficient (CC) or the area under the Receiver Operating Characteristic (ROC) curve (AUC) provide more useful measures than accuracy in assessing the performance of the classifiers [26]. As shown in Table 5, the observed performance of the three prediction methods on HLA-DRB1*0301 MHCBN-UPDS version of this dataset appears to be overly optimistic relative to that on its *similarity-reduced* and *weighted* counterparts. Interestingly, the 5-spectrum kernel is competitive with CTD and LA on the MHCBN-UPDS dataset, whereas its performance on MHCBN-SRDS1 and MHCBN-SRDS2 is much worse than that of the CTD and the LA classifiers.

**Table 5:** Performance of prediction methods on MHCBN HLA-DRB1*0301 *unique*, *similarity-reduced*, and *weighted* datasets using 5-fold cross-validation test.

Dataset	Method	ACC	Sn	Sp	CC	AUC
UPDS	CTD	72.51	73.95	70.87	0.448	0.787
	LA	71.89	73.56	70.00	0.436	0.795
	5-spectrum	70.26	82.76	56.09	0.405	0.770
SRDS1	CTD	63.41	64.23	62.67	0.269	0.661
	LA	58.54	59.85	57.33	0.172	0.617
	5-spectrum	42.16	63.50	22.67	−0.152	0.323
SRDS2	CTD	59.93	59.06	60.69	0.197	0.628
	LA	55.88	54.33	57.24	0.116	0.563
	5-spectrum	35.29	37.01	33.79	−0.292	0.273
SRDS3	CTD	64.62	60.57	67.91	0.285	0.675
	LA	67.18	61.14	72.09	0.334	0.736
	5-spectrum	63.08	49.71	73.95	0.244	0.678
WUPDS	CTD	65.27	61.04	68.88	0.300	0.678
	LA	66.66	64.47	68.53	0.330	0.710
	5-spectrum	59.97	58.70	61.04	0.197	0.648

Our results also demonstrate that conclusions of superior performance of one method relative to another that are based on estimates of performance obtained using UPDS versions of MHC-II benchmark datasets can be misleading. For example, from results shown in Tables 4 and 5, one might be tempted to conclude that predictors that use the 5-spectrum kernel are competitive with those that use CTD representation and the LA kernel. However, the 5-spectrum kernel is outperformed by CTD and LA on the *similarity-reduced* datasets. Similarly, conclusions drawn from experiments using the UPDS datasets (Tables 4 and 5) regarding the performance of the CTD and the LA kernel classifiers are contradicted by the their observed performance on the corresponding *similarity-reduced* datasets SRDS1 and SRDS2.

### Limitations of the MHCBench benchmark data

Comparison of SRDS1, SRDS2, and SRDS3 versions of the datasets used in this study reveals an important limitation of the MHCBench dataset which is a widely used benchmark for comparing MHC-II binding peptide prediction methods.

**Figure 1:**
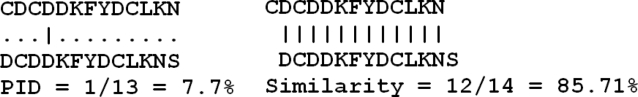
Example of two peptides from MHCBN-SRDS3 HLA-DRB1*0301 dataset. Although the two peptides share 85.71% sequence similarity, the computed percent identity (PID) used to define the similarity between these two peptides in MHCBench benchmark is only 7.7%.

Recall that the SRDS3 versions of our datasets are derived using the same procedure that was used in MHCBench to generate *similarity-reduced* datasets. It is clear from the data summarized in Tables 1–3 that the size of a SRDS3 version of a dataset is: often larger than the size of its SRDS2 counterpart, and sometimes larger than the size of its SRDS1 counterpart. Closer examination of the peptides in SRDS3 datasets reveals that SRDS3 datasets may contain several highly similar peptides (e.g., peptides with more than 80% sequence similarity). This is illustrated by the example shown in Figure 1: the two peptides in the SRDS3 version of the HLA-DRB1*0301 dataset share overall sequence similarity of 85.71%. However, the procedure used to construct *similarity-reduced* MHCBench dataset will keep both of these peptides in the resulting dataset because the computed percent identity (PID) between the two peptides is only 7.7%, well below the threshold of 80% PID used to identify similar peptides in MHCBench [20]. Thus, the similarity reduction procedure used in MHCBench dataset (which relies on a strict gapless alignment) may not eliminate all highly similar peptides.

The preceding observation explains why the number of peptides in the SRDS3 versions of the datasets is usually greater than that in SRDS1 and SRDS2 datasets (see Tables 1–3). More importantly, because of the presence of a number of highly similar peptides in some SRDS3 datasets, the observed performance of the three prediction methods on the SRDS3 datasets may be overly optimistic relative to that estimated from their SRDS1 and SRDS2 counterparts. Because the classifier using the 5-spectrum kernel in fact relies on the degree of (gapless) match between a sequence pattern present in one or more training sequences and a test sequence, it benefits from the presence of a high degree of similarity between a test sequence and one or more training sequences in ways that the other two classifiers do not. Consequently classifiers that use the 5-spectrum kernel can appear to be competitive with, and perhaps even outperform those that use the CTD representation or the LA kernel when their performance is compared using SRDS3 datasets (and for similar reasons, the MHCBench benchmark data).

### Comparison of the CTD, LA, and the k-spectrum kernel methods

In machine learning and bioinformatics literature, claims of superiority of one method over another are often based on the outcome of suitable statistical tests. Hence it is interesting to examine the differences in the conclusions obtained when statistical tests are used to compare the performance of prediction methods based on the empirical estimates of their performance on the UPDS, SRDS1, SRDS2, SRDS3, and WPDS versions of the datasets.

Several non-parametric statistical tests [27], [28] have been recently recommended for comparing different classifiers on multiple datasets (accounting for the effects of multiple comparisons) [29]. In our analysis, we apply a three-step procedure proposed by Demšar [29]. First, the classifiers to be compared are ranked on the basis of their observed performance (e.g., AUC) on each dataset. Second, the Friedman test is applied to determine whether the measured average ranks are significantly different from the mean rank under the null hypothesis. Third, if the null hypothesis can be rejected at a significance level of 0.05, the Nemenyi test is used to determine whether significant differences exist between any given pair of classifiers.

### Statistical analysis of results on the MHCPEP datasets

Tables 6–10 compare the AUC of the three prediction methods on the five versions of the MHCPEP datasets. For each dataset, the rank of each classifier is shown in parentheses. The last row in each table summarizes the average AUC and rank for each classifier. Demšar [29] has suggested that the average ranks by themselves provide a reasonably fair comparison of classifiers. Interestingly, the LA kernel has the *worst* rank among the three methods when the comparison is based on the observed performance on the UPDS datasets, whereas it has the *best* rank among the three methods when the comparison is based on the *similarity-reduced* or the *weighted* datasets. Tables 6–10 also show that the rank of the 5-spectrum kernel is competitive with that of CTD on UPDS and SRDS3. This observation is consistent with the presence of a number of highly similar sequences in SRDS3 datasets.

**Table 6:** AUC values for the three methods evaluated on MHCPEP-UPDS datasets.

Allele	5-spectrum	LA	CTD
HLA-DQ2	0.908(2)	0.905(3)	0.939(1)
HLA-DQ4	0.628(3)	0.903(2)	0.934(1)
HLA-DQ7	0.856(2)	0.860(1)	0.853(3)
HLA-DR1	0.883(1)	0.872(2)	0.863(3)
HLA-DR2	0.884(1)	0.866(2)	0.829(3)
HLA-DR3	0.854(3)	0.869(1)	0.862(2)
HLA-DR4	0.917(1)	0.862(3)	0.906(2)
HLA-DR5	0.905(1)	0.864(3)	0.887(2)
HLA-DR7	0.916(1)	0.858(3)	0.904(2)
HLA-DR8	0.894(3)	0.896(2)	0.903(1)
HLA-DR9	0.836(3)	0.880(2)	0.913(1)
HLA-DR11	0.910(3)	0.938(2)	0.958(1)
HLA-DR13	0.875(3)	0.905(2)	0.920(1)
HLA-DR15	0.887(1)	0.829(3)	0.867(2)
HLA-DR17	0.907(2)	0.907(2)	0.934(1)
HLA-DR51	0.924(1)	0.891(2)	0.886(3)
I-Ab	0.865(2)	0.855(3)	0.875(1)
I-Ad	0.942(1)	0.898(3)	0.902(2)
I-Ag7	0.916(1)	0.896(2)	0.887(3)
I-Ak	0.909(1)	0.872(3)	0.881(2)
I-Ed	0.918(3)	0.921(2)	0.936(1)
I-Ek	0.934(3)	0.940(2)	0.951(1)
Average	0.885(1.91)	0.886(2.27)	0.900(1.77)

For each dataset, the rank of each classifier is shown in parentheses.

**Table 7 pone-0003268-t007:** AUC values for the three methods evaluated on MHCPEP-SRDS1 datasets.

Allele	5-spectrum	LA	CTD
HLA-DQ2	0.789(3)	0.852(2)	0.853(1)
HLA-DQ4	0.544(3)	0.854(2)	0.881(1)
HLA-DQ7	0.677(3)	0.799(1)	0.726(2)
HLA-DR1	0.662(3)	0.801(1)	0.744(2)
HLA-DR2	0.694(3)	0.795(1)	0.781(2)
HLA-DR3	0.603(2)	0.678(1)	0.572(3)
HLA-DR4	0.710(3)	0.751(1)	0.723(2)
HLA-DR5	0.691(3)	0.776(2)	0.784(1)
HLA-DR7	0.721(2)	0.702(3)	0.732(1)
HLA-DR8	0.552(3)	0.625(2)	0.694(1)
HLA-DR9	0.620(3)	0.746(1)	0.721(2)
HLA-DR11	0.703(3)	0.912(1)	0.890(2)
HLA-DR13	0.746(3)	0.827(2)	0.837(1)
HLA-DR15	0.711(2)	0.718(1)	0.667(3)
HLA-DR17	0.789(3)	0.806(2)	0.876(1)
HLA-DR51	0.651(2)	0.788(1)	0.603(3)
I-Ab	0.620(3)	0.705(1)	0.680(2)
I-Ad	0.787(3)	0.818(1)	0.804(2)
I-Ag7	0.718(2)	0.778(1)	0.702(3)
I-Ak	0.761(3)	0.800(1)	0.796(2)
I-Ed	0.826(3)	0.903(2)	0.932(1)
I-Ek	0.874(3)	0.913(2)	0.941(1)
Average	0.702(2.77)	0.789(1.45)	0.770(1.77)

For each dataset, the rank of each classifier is shown in parentheses.

**Table 8 pone-0003268-t008:** AUC values for the three methods evaluated on MHCPEP-SRDS2 datasets.

Allele	5-spectrum	LA	CTD
HLA-DQ2	0.566(3)	0.678(1)	0.573(2)
HLA-DQ4	0.590(3)	0.954(1)	0.817(2)
HLA-DQ7	0.616(3)	0.713(1)	0.709(2)
HLA-DR1	0.562(3)	0.715(1)	0.711(2)
HLA-DR2	0.548(3)	0.633(1)	0.614(2)
HLA-DR3	0.514(3)	0.602(1)	0.572(2)
HLA-DR4	0.578(3)	0.711(1)	0.692(2)
HLA-DR5	0.583(3)	0.622(2)	0.625(1)
HLA-DR7	0.562(3)	0.622(1)	0.599(2)
HLA-DR8	0.526(3)	0.717(1)	0.680(2)
HLA-DR9	0.488(3)	0.754(1)	0.690(2)
HLA-DR11	0.528(3)	0.810(1)	0.792(2)
HLA-DR13	0.518(3)	0.827(1)	0.587(2)
HLA-DR15	0.592(3)	0.698(1)	0.689(2)
HLA-DR17	0.568(2)	0.612(1)	0.550(3)
HLA-DR51	0.578(3)	0.664(1)	0.595(2)
I-Ab	0.570(3)	0.624(2)	0.638(1)
I-Ad	0.623(2)	0.700(1)	0.618(3)
I-Ag7	0.713(2)	0.756(1)	0.632(3)
I-Ak	0.586(3)	0.664(1)	0.661(2)
I-Ed	0.645(3)	0.760(1)	0.744(2)
I-Ek	0.606(3)	0.756(1)	0.703(2)
Average	0.575(2.86)	0.709(1.09)	0.659(2.05)

For each dataset, the rank of each classifier is shown in parentheses.

**Table 9 pone-0003268-t009:** AUC values for the three methods evaluated on MHCPEP-SRDS3 datasets.

Allele	5-spectrum	LA	CTD
HLA-DQ2	0.663(2)	0.655(3)	0.754(1)
HLA-DQ4	0.608(3)	0.900(1)	0.900(1)
HLA-DQ7	0.699(3)	0.757(1)	0.706(2)
HLA-DR1	0.676(3)	0.747(1)	0.720(2)
HLA-DR2	0.724(2)	0.736(1)	0.686(3)
HLA-DR3	0.623(2)	0.657(1)	0.532(3)
HLA-DR4	0.679(3)	0.717(1)	0.695(2)
HLA-DR5	0.719(2)	0.723(1)	0.617(3)
HLA-DR7	0.631(2)	0.765(1)	0.613(3)
HLA-DR8	0.608(3)	0.732(1)	0.714(2)
HLA-DR9	0.520(3)	0.779(2)	0.792(1)
HLA-DR11	0.544(3)	0.854(1)	0.850(2)
HLA-DR13	0.563(3)	0.623(2)	0.630(1)
HLA-DR15	0.805(1)	0.713(2)	0.663(3)
HLA-DR17	0.629(3)	0.769(1)	0.682(2)
HLA-DR51	0.800(1)	0.780(2)	0.672(3)
I-Ab	0.606(3)	0.611(2)	0.618(1)
I-Ad	0.821(1)	0.785(2)	0.676(3)
I-Ag7	0.823(1)	0.804(2)	0.757(3)
I-Ak	0.768(1)	0.766(2)	0.691(3)
I-Ed	0.828(2)	0.852(1)	0.787(3)
I-Ek	0.714(2)	0.789(1)	0.699(3)
Average	0.684(2.23)	0.751(1.45)	0.702(2.27)

For each dataset, the rank of each classifier is shown in parentheses.

**Table 10:** AUC values for the three methods evaluated on MHCPEP-WUPDS datasets.

Allele	5-spectrum	LA	CTD
HLA-DQ2	0.717(3)	0.738(2)	0.772(1)
HLA-DQ4	0.543(3)	0.882(2)	0.925(1)
HLA-DQ7	0.716(3)	0.786(2)	0.812(1)
HLA-DR1	0.696(3)	0.710(1)	0.699(2)
HLA-DR2	0.682(2)	0.688(1)	0.617(3)
HLA-DR3	0.612(3)	0.678(1)	0.614(2)
HLA-DR4	0.723(2.5)	0.723(2.5)	0.730(1)
HLA-DR5	0.765(1)	0.709(3)	0.733(2)
HLA-DR7	0.714(1)	0.599(3)	0.632(2)
HLA-DR8	0.796(3)	0.810(1)	0.802(2)
HLA-DR9	0.806(2)	0.819(1)	0.738(3)
HLA-DR11	0.612(3)	0.798(2)	0.830(1)
HLA-DR13	0.620(2)	0.714(1)	0.605(3)
HLA-DR15	0.760(1)	0.627(2)	0.587(3)
HLA-DR17	0.747(2)	0.760(1)	0.679(3)
HLA-DR51	0.838(1)	0.786(2)	0.718(3)
I-Ab	0.650(2)	0.669(1)	0.636(3)
I-Ad	0.815(1)	0.740(2)	0.707(3)
I-Ag7	0.820(1)	0.797(2)	0.700(3)
I-Ak	0.778(1)	0.684(2)	0.680(3)
I-Ed	0.742(3)	0.760(2)	0.805(1)
I-Ek	0.734(3)	0.824(1)	0.805(2)
Average	0.722(2.11)	0.741(1.7)	0.719(2.18)

For each dataset, the rank of each classifier is shown in parentheses.

**Figure 2:**
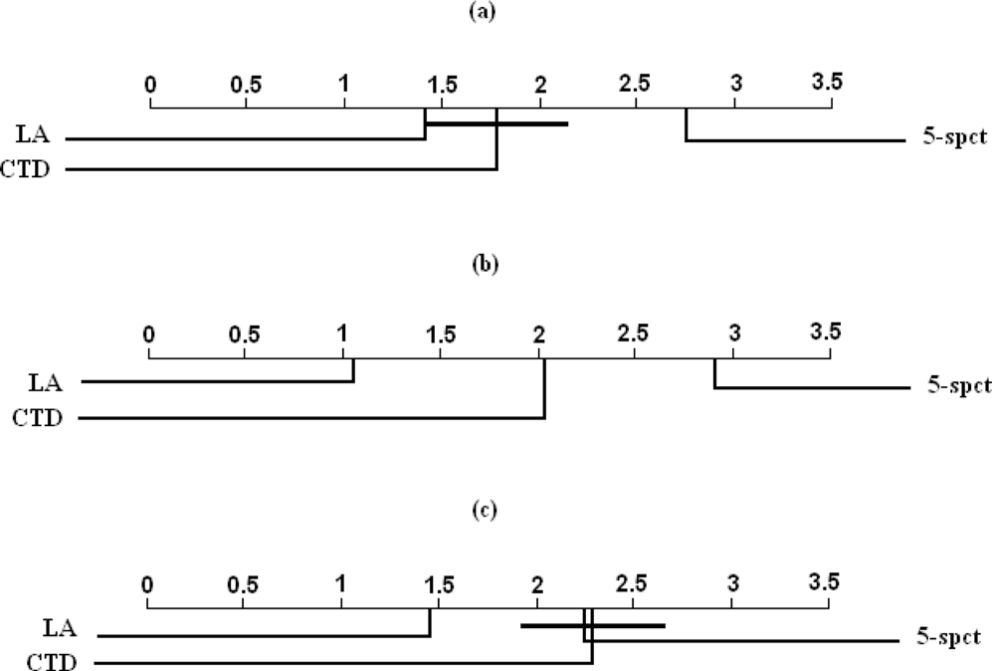
Pair-wise comparisons of classifiers with the Nemenyi test applied to results on a) MHCPEP-SRDS1, b) MHCPEP-SRDS2, and c) MHCPEP-SRDS3. Classifiers that are not significantly different (at p-value = 0.05) are connected.

To determine whether the differences in average ranks are statistically significant, we applied the Friedman test [29] to the rank data in Tables 6–10. At significance level of 0.05, the Friedman test did not indicate a statistically significant difference between the methods on the UPDS and WUPDS datasets. However, in the case of the *similarity-reduced* datasets, the Friedman test indicated statistically significant differences between the methods being compared. Thus, we conclude that the three methods are competitive with each other on the UPDS and WUPDS datasets, and that there is at least one pair of classifiers with significant difference in performance on the three versions of *similarity-reduced* datasets. Furthermore, for each version of MHCPEP *similarity-reduced* datasets, the Nemenyi test was applied to determine whether significant differences exist between any given pair of classifiers. Figure 2 summarizes the results of the pair-wise comparisons performed using the Nemenyi test. We find that on the SRDS1 versions of the datasets, both the LA and the CTD methods significantly outperform the 5-spectrum kernel and that there are no statistically significant differences between the LA kernel and the CTD classifier. On SRDS2 datasets, we find that, the performance of each of the three methods is significantly different from that of the other two methods, with the LA and the CTD methods ranked first and second, respectively. On SRDS3 datasets, we observe that the performance of the LA kernel is significantly better than that of the CTD and the 5-spectrum classifiers, with no significant differences between the CTD and the 5-spectrum classifiers.

### Statistical analysis of results on the MHCBN and the IEDB datasets

**Figure 3:**
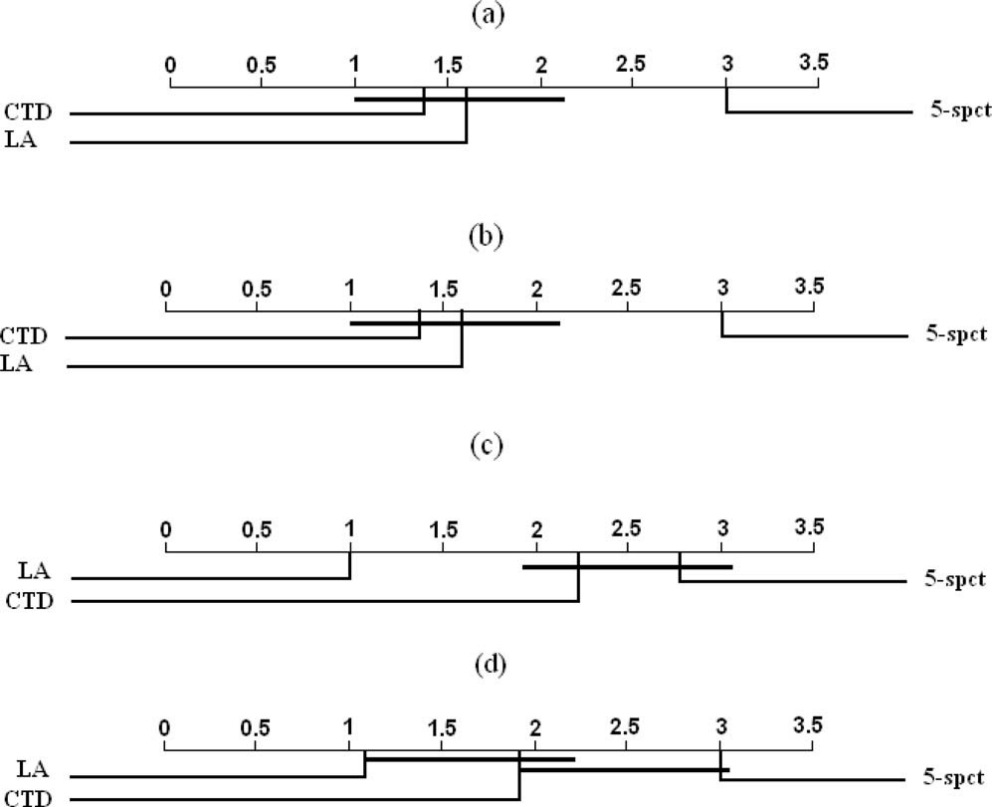
Pair-wise comparisons of classifiers with the Nemenyi test applied to results on a) MHCBN-SRDS1, b) MHCBN-SRDS2, c) MHCBN-SRDS3, and d) MHCBN-WUPDS. Classifiers that are not significantly different (at p-value = 0.05) are connected.

We summarize the results of applying Demšar's three-step procedure to the results obtained on the five versions of MHCBN and IEDB datasets, respectively. In the case of the MHCBN datasets, Tables 11–15 show the estimated AUC and rank of each classifier on each dataset. The results of the Freidman test (at a significance level of 0.05) applied to the results in each table did not indicate significant differences in performance among the CTD, the LA, and the 5-spectrum kernel classifiers on the UPDS dataset. However, the test indicated statistically significant differences among the methods in the case of the SRDS1, SRDS2, SRDS3, and the WUPDS datasets. Figure 3 summarizes the results of the pair-wise comparisons using the Nemenyi test. In the case of the SRDS1 and the SRDS2 datasets, we find that the performance of both the LA kernel and the CTD classifiers is significantly better than that of the 5-spectrum kernel classifier and that there are no significant differences between the LA kernel and the CTD classifiers. In the case of the SRDS3 datasets, we find that the performance of the LA kernel classifier is significantly better than that of the CTD and the 5-spectrum classifiers, and that no significant differences exist between the CTD and the 5-spectrum classifiers. In the case of the WUPDS datasets, we find that the LA kernel classifier significantly outperforms the 5-spectrun kernel and that there are no significant differences between the LA and the CTD and between the CTD and the 5-spectrum classifiers.

**Table 11:** AUC values for the three methods evaluated on MHCBN-UPDS datasets.

Allele	5-spectrum	LA	CTD
HLA-DR1	0.747(3)	0.768(2)	0.789(1)
HLA-DR2	0.806(1)	0.771(3)	0.786(2)
HLA-DR5	0.743(3)	0.748(2)	0.752(1)
HLA-DRB10101	0.758(3)	0.799(2)	0.804(1)
HLA-DRB10301	0.770(3)	0.795(1)	0.787(2)
HLA-DRB10401	0.705(3)	0.780(1)	0.721(2)
HLA-DRB10701	0.778(2)	0.842(1)	0.732(3)
HLA-DRB11101	0.754(3)	0.874(1)	0.832(2)
Average	0.758(2.63)	0.797(1.63)	0.775(1.75)

For each dataset, the rank of each classifier is shown in parentheses.

**Table 12 pone-0003268-t012:** AUC values for the three methods evaluated on MHCBN-SRDS1 datasets.

Allele	5-spectrum	LA	CTD
HLA-DR1	0.545(3)	0.784(1)	0.738(2)
HLA-DR2	0.456(3)	0.707(2)	0.750(1)
HLA-DR5	0.533(3)	0.657(2)	0.692(1)
HLA-DRB10101	0.456(3)	0.690(2)	0.748(1)
HLA-DRB10301	0.323(3)	0.617(2)	0.661(1)
HLA-DRB10401	0.381(3)	0.676(1)	0.655(2)
HLA-DRB10701	0.424(3)	0.665(2)	0.748(1)
HLA-DRB11101	0.493(3)	0.776(1)	0.759(2)
Average	0.451(3.00)	0.697(1.63)	0.719(1.38)

For each dataset, the rank of each classifier is shown in parentheses.

**Table 13 pone-0003268-t013:** AUC values for the three methods evaluated on MHCBN-SRDS2 datasets.

Allele	5-spectrum	LA	CTD
HLA-DR1	0.448(3)	0.717(1)	0.698(2)
HLA-DR2	0.374(3)	0.665(2)	0.716(1)
HLA-DR5	0.369(3)	0.459(2)	0.588(1)
HLA-DRB10101	0.351(3)	0.705(1)	0.683(2)
HLA-DRB10301	0.273(3)	0.563(2)	0.628(1)
HLA-DRB10401	0.261(3)	0.658(1)	0.620(2)
HLA-DRB10701	0.414(3)	0.617(2)	0.696(1)
HLA-DRB11101	0.386(3)	0.705(2)	0.757(1)
Average	0.360(3.00)	0.636(1.63)	0.673(1.38)

For each dataset, the rank of each classifier is shown in parentheses.

**Table 14 pone-0003268-t014:** AUC values for the three methods evaluated on MHCBN-SRDS3 datasets.

Allele	5-spectrum	LA	CTD
HLA-DR1	0.685(3)	0.768(1)	0.743(2)
HLA-DR2	0.709(3)	0.741(1)	0.719(2)
HLA-DR5	0.557(3)	0.616(1)	0.608(2)
HLA-DRB10101	0.691(3)	0.819(1)	0.725(2)
HLA-DRB10301	0.678(2)	0.736(1)	0.675(3)
HLA-DRB10401	0.624(3)	0.760(1)	0.710(2)
HLA-DRB10701	0.737(2)	0.794(1)	0.671(3)
HLA-DRB11101	0.755(3)	0.816(1)	0.775(2)
Average	0.680(2.75)	0.756(1.00)	0.703(2.25)

For each dataset, the rank of each classifier is shown in parentheses.

**Table 15:** AUC values for the three methods evaluated on MHCBN-WUPDS datasets.

Allele	5-spectrum	LA	CTD
HLA-DR1	0.655(3)	0.747(1)	0.732(2)
HLA-DR2	0.636(3)	0.717(2)	0.740(1)
HLA-DR5	0.518(3)	0.594(1)	0.543(2)
HLA-DRB10101	0.535(3)	0.672(1)	0.666(2)
HLA-DRB10301	0.648(3)	0.710(1)	0.678(2)
HLA-DRB10401	0.536(3)	0.757(1)	0.701(2)
HLA-DRB10701	0.667(3)	0.724(1)	0.702(2)
HLA-DRB11101	0.676(3)	0.820(1)	0.789(2)
Average	0.609(3)	0.718(1.13)	0.694(1.88)

For each dataset, the rank of each classifier is shown in parentheses.

**Figure 4:**
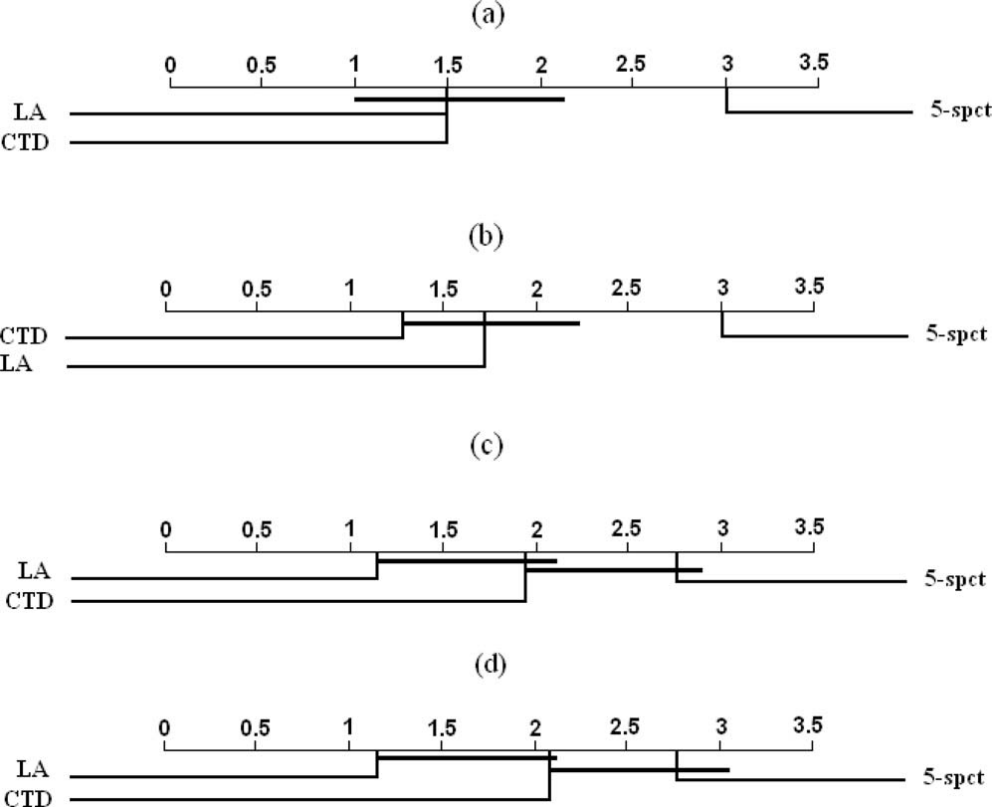
Pair-wise comparisons of classifiers with the Nemenyi test applied to results on a) IEDB-SRDS1, b) IEDB-SRDS2, c) IEDB-SRDS3, and d) IEDB-WUPDS. Classifiers that are not significantly different (at p-value = 0.05) are connected.

Results of Demšar's statistical test applied to the IEDB datasets are shown in Tables S46–S50 (Data S1 in supporting information) and Figure 4. As in the case of MHCPEP and MHCBN, we see no significant differences in the performance of different classifiers on IEDB-UPDS datasets. However, in the case of the other datasets, we find at least one pair of classifiers with significant differences in performance. As shown in Figure 4, both the LA and the CTD classifiers significantly outperform the 5-spectrum classifier on the SRDS1 and the SRDS2 versions of the IEDB datasets. However, no significant differences are observed between the CTD and the 5-spectrum methods on the SRDS3 and WUPDS versions of the IEDB datasets.

### Performance on the blind test set

The results summarized above underscore the importance of *similarity-reduced* MHC-II datasets for obtaining a realistic estimation of the classifier performance and avoiding misleading conclusions. However, one might argue that in practice, when developers of MHC-II binding peptide prediction methods make an implementation of their methods publicly available (e.g., as an online web server or as a web service), it might be better to utilize as much of the available data as possible to train the predictor. Hence, it is interesting to explore whether the UPDS datasets should be preferred over the *similarity-reduced* counterparts to avoid any potential loss of useful information due to the elimination of highly similar peptides in a setting where the goal is to optimize the predictive performance of the classifier on novel peptides. In what follows, we attempt to answer this question using five allele-specific blind test sets [30] to evaluate the performance of the three prediction methods trained on the *unique*, *similarity-reduced*, and *weighted* versions of the MHCBN data for the corresponding alleles.

Table 16 shows that the 5-spectrum kernel classifier consistently performs poorly (*AUC*≈0.5) on the allele-specific blind test sets regardless of the version of the MHCBN dataset used for training the classifier. This finding is consistent with the cross-validation performance estimates obtained on the MHCBN SRDS1 and SRDS2 datasets (see Tables 12 and 13).

**Table 16:** AUC values for 5-spectrum based classifiers trained using MHCBN- UPDS, SRDS1, SRDS2, SRDS3, and WUPDS datasets and evaluated on the blind test sets of Wang et al. [30].

Allele	UPDS	SRDS1	SRDS2	SRDS3	WUPDS
HLA-DRB1-0101	0.505	0.503	0.504	0.506	0.505
HLA-DRB1-0301	0.518	0.515	0.515	0.516	0.518
HLA-DRB1-0401	0.504	0.500	0.500	0.501	0.487
HLA-DRB1-0701	0.500	0.500	0.500	0.500	0.496
HLA-DRB1-1101	0.500	0.500	0.500	0.500	0.496
Average	0.505	0.504	0.504	0.505	0.500

Table 17 shows the performance on the blind test sets of the CTD classifiers trained on different versions of MHCBN datasets. Interestingly, the CTD classifiers appear to be relatively insensitive to the choice of the specific version of the MHCBN dataset on which they were trained, with an average AUC≈0.66 in each case.

**Table 17:** AUC values for CTD classifiers trained using MHCBN- UPDS, SRDS1, SRDS2, SRDS3, and WUPDS datasets and evaluated on the blind test sets of Wang et al. [30].

Allele	UPDS	SRDS1	SRDS2	SRDS3	WUPDS
HLA-DRB1-0101	0.689	0.707	0.684	0.714	0.629
HLA-DRB1-0301	0.595	0.589	0.597	0.596	0.585
HLA-DRB1-0401	0.605	0.584	0.611	0.633	0.601
HLA-DRB1-0701	0.675	0.711	0.699	0.684	0.694
HLA-DRB1-1101	0.732	0.701	0.719	0.713	0.735
Average	0.659	0.658	0.662	0.668	0.649

Finally, Table 18 summarizes the performance on the blind test sets of the LA classifiers trained on the different versions of MHCBN datasets. Interestingly, the best performance (on four out of the five allele-specific blind test sets) is observed in the case of the LA classifiers trained on the SRDS2 versions of the corresponding allele-specific datasets.

**Table 18:** AUC values for LA classifiers trained using MHCBN- UPDS, SRDS1, SRDS2, SRDS3, and WUPDS datasets and evaluated on the blind test sets of Wang et al. [30].

Allele	UPDS	SRDS1	SRDS2	SRDS3	WUPDS
HLA-DRB1-0101	0.675	0.650	0.756	0.736	0.703
HLA-DRB1-0301	0.604	0.647	0.651	0.637	0.604
HLA-DRB1-0401	0.554	0.548	0.610	0.595	0.573
HLA-DRB1-0701	0.627	0.692	0.692	0.677	0.627
HLA-DRB1-1101	0.775	0.722	0.701	0.730	0.775
Average	0.647	0.652	0.682	0.675	0.656

In summary, our results show that MHC-II predictors trained on the similarity reduced versions of the dataset generally outperform those trained on the UPDS dataset. This suggests that similarity reduction contributes to improved generalization on blind dataset.

## Discussion

### Related work

Several previous studies have considered the importance of similarity reduction in datasets of MHC-II peptides. MHCBench [20] is a benchmark of eight HLA-DRB1*0401 datasets representing a set of *unique* peptides (Set1), a dataset of natural peptides (Set2, derived from Set1 by removing peptides with >75% Alanine residues), two non-redundant datasets (Set3a and Set3b derived from Set1 and Set2, respectively), two balanced datasets (Set4a and Set4b derived from Set1 and Set2 by randomly selecting equal numbers of binding and non-binding peptides), and two recent datasets of ligands (Set5a and Set5b, derived from Set1 and Set2 by considering only the most recently reported peptides). However, this benchmark considers only a single MHC-II allele, namely, HLA-DR4 (B1*0401). More importantly, as shown by our analysis of SRDS3 datasets, the similarity reduction procedure used in MHCBench is not stringent enough to ensure elimination of highly similar peptides.

Nielsen et al. [6] and Murugan et al. [18] trained their classifiers using data extracted from MHCPEP and SYFPETHI databases and evaluated the classifiers using ten test sets, from which peptides similar to peptides in the training datasets had been removed. Recently, Nielsen et al. [7] presented an MHC-II benchmarking dataset for regression tasks: each peptide is labeled with a real value indicating the binding affinity of the peptide. In this benchmark dataset, each set of allele-specific data had been partitioned into five subsets with minimal sequence overlap. However, neither of these studies explicitly examined the limitations of widely used benchmark datasets or the full implications of using MHC-II datasets of unique peptides in evaluating alternative methods.

Mallios [31] compared three HLA-DRB1*0101 and HLA-DRB1*0401 prediction tools using an independent test set of two proteins. A consensus approach combining the predictions of the three methods was shown to be superior to the three methods. However, the significance of this result is limited by the small dataset utilized in this study.

Two recent studies [30], [32] have pointed out some of the limitations of existing MHC-II prediction methods in identifying potential MHC-II binding peptides. Gowthaman et al. [32] used 179 peptides derived from eight antigens and covering seven MHC-II alleles to evaluate the performance of six commonly used MHC-II prediction methods and concluded that none of these methods can reliably identify potential MHC-II binding peptides. Wang et al. [30] introduced a large benchmark dataset of previously unpublished peptides and used it to assess the performance of nine publicly available MHC-II binding peptide prediction methods. Both studies showed that the predictive performance of existing MHC-II prediction tools on independent blind test sets is substantially worse than the performance of these tools reported by their developers. Our work complements these studies by providing a plausible explanation of this result.

We have shown that the previously reported similarity reduction methods may not eliminate highly similar peptides, i.e., peptides that share >80% sequence identity still *pass* the similarity test. We have proposed a two-step similarity reduction procedure that is much more stringent than those currently in use for similarity reduction with MHC-II benchmark datasets. We have used the similarity reduction method used in MHCBench, as well as our proposed 2-stage method to derive *similarity-reduced* MHC-II benchmark datasets based on peptides retrieved from MHCPEP and MHCBN databases. Comparison of the *similarity-reduced* versions of MHCPEP, MHCBN, and IEDB datasets with their original UPDS counterparts showed that nearly 50% of the peptides in the UPDS datasets are, in fact, highly similar.

### Extensions to multi-class and multi-label prediction problems

Our description of the proposed similarity reduction procedure assumes a 2-class prediction problem. However, our proposed approach can easily be adapted to multi-class prediction (wherein an instance has associated with one of several mutually exclusive labels). One can simply apply the similarity reduction procedure separately to data from each class.

A more interesting setting is that of multi-label prediction (wherein each instance is associated with a subset of a set of candidate labels). Consider for example, the problem of predicting promiscuous MHC binding peptides [33], where each peptide can bind to multiple HLA molecules. Current methods for multi-label prediction typically reduce the multi-label prediction task to a collection of binary prediction tasks [34]. Hence, the similarity reduction methods proposed in this paper can be directly applied to the binary labeled datasets resulting from such a reduction.

### Implications for rigorous assessment of MHC-II binding peptide prediction methods

The results of our study show that the observed performance of some of the methods (e.g., the CTD and the LA kernels) on benchmark datasets of *unique* peptides can be rather optimistic relative to the performance of the same methods on *similarity-reduced* counterparts of the same datasets or on blind test sets. This suggests that the performance of existing MHC-II prediction methods, when applied to novel peptide sequences, may turn out to be less satisfactory than one might have been led to believe based on the reported performance of such methods on some of the widely used benchmark. Moreover, the conclusions based on observed performance on datasets of *unique* peptides regarding the superior performance of one method relative to another can be highly unreliable in more realistic settings e.g., predictions of novel peptides.

These results underscore the importance of rigorous comparative evaluation of a broad range of existing methods for MHC-II binding peptides prediction methods using *similarity-reduced* datasets. We expect that such studies are likely to show much greater room for improvement over the state-of-the-art MHC-II prediction tools than one might be led to believe based on reported performance on the widely-used benchmark datasets and motivate the research community to develop improved methods for this important task. We hope that such comparisons will be facilitated by the availability of the *similarity-reduced* versions of MHCPEP, MHCBN, and IEDB datasets used in our experiments. These datasets (Datasets S1, S2 and S3), Java source code implementation of the similarity reduction and weighting procedures (Code S1), and the supplementary materials (Data S1) have been made freely available (see Supporting Information).

## Materials and Methods

The datasets used in this study are derived from MHCPEP [21], MHCBN [22], and IEDB [23], which are manually curated repositories of MHC binding peptides reported in the literature.

We extracted 22 MHC-II allele datasets (each with at least 100 binders) from the MHCPEP database. Because MHCPEP contains only MHC-II binding peptides (“positive examples”), for each allele, we generated an equal number of non-binders (“negative examples”) by randomly extracting protein fragments from SwissProt [35] protein sequences such that: (i) The length distribution of negative examples is identical to that of the positive examples; (ii) None of the non-binding peptides appear in the set of binders.

Unlike MHCPEP, MHCBN is a database of binding and non-binding MHC peptides. MHCBN version 4.0 has 35 MHC-II alleles with at least 100 binders. Out of these 35 alleles, only eight alleles have at least 100 non-binders. We extracted the MHCBN benchmark dataset used in this study from the alleles for which at least 100 binders and non-binders peptides are available in MHCBN.

The Immune Epitope Database and Analysis Resource (IEDB) [23] is a rich resource of MHC binding data curated from the literature or submitted by immunologists. For each reported peptide, IEDB provides qualitative (i.e., Negative or Positive) and quantitative (i.e., IC50) measurements whenever available. We used both qualitative and quantitative measurements for constructing 12 HLA binary labeled datasets as follows:

- Peptides with no reported quantitative measurements are discarded.
- Peptides with “Positive” qualitative measurement and quantitative measurement less than 500 nM are classified as binders.
- Peptides with “Positive” qualitative measurement and quantitative measurement greater than or equal 500 nM are classified as non-binders.
- Peptides with “Negative” qualitative measurement and quantitative measurement greater than or equal 500 nM are classified as non-binders.
- Peptides with “Negative” qualitative measurement and quantitative measurement less than 500 nM are discarded.

The reported MHC binding sites are typically identified using truncation, substitution, or mutations in a base peptide [36]. Because different reported MHC-II binding peptides might actually correspond to experimental manipulation of the same MHC-II binding region using different experimental techniques or different choices of amino acids targeted for truncation, substitution, or mutation, it is not surprising that that MHC databases contain a significant number of highly similar peptides. Hence, we used several similarity reduction methods to extract several different versions of the dataset from each set of sequences.

It should be noted that the existence of highly similar peptides belonging to the same category may result in an over-optimistic estimation of the classifier performance. Therefore, we applied the similarity reduction procedures separately to the set of binders and non-binders in each dataset. The following sections describe the similarity reduction procedures and the resulting *similarity-reduced* datasets.

### Similarity reduction procedures

**Figure 5:**
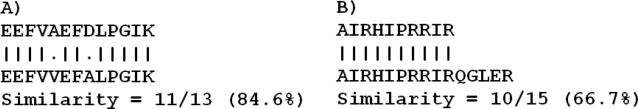
Two types of similar peptides that frequently appear in MHC databases.

An example of two different types of similar peptides that frequently occur in MHC peptides databases is shown in Figure 5. In type I, two peptides differ from each other in terms of only one or two amino acids (see Figure 5A). Such highly similar peptides are likely to have come from different mutation experiments targeting different sites of the same MHC-II binding peptide. For example, Garcia et al. [37] report an HLA-DRB1*0401 binding peptide (WGENDTDVFVLNNTR) and 12 additional binding peptides derived from that peptide by replacing one of the amino acid in (WGENDTDVFVLNNTR) sequence with Glycine and experimentally determining the binding affinity of the new peptide. In type II, we find that a shorter peptide in one allele dataset corresponds to a sub-sequence of a longer one that is also in the allele dataset (see Figure 5B).

Standard approaches to identifying similar peptide sequences rely on the use of a sequence similarity threshold. Sequences that are within a certain predetermined similarity threshold relative to a target sequence are eliminated from the dataset. However, the use of such a simple approach to obtaining a similarity reduced dataset is complicated by the high degree of variability in the length of MHC-II peptides. Using a single fixed similarity cutoff value (e.g. 80%) might not be effective in eliminating type II similar peptides. On the other hand, an attempt to eliminate one of the two such similar sequences by using of a more stringent similarity threshold could result in elimination of most of the dataset.

To address this problem, we used a two-step similarity reduction procedure to eliminate similar peptides of types I and II:

- Step 1 eliminates similar peptides based on a criterion proposed by Nielsen et al. [7]. Two peptides are considered similar if they share a 9-mer subsequence. This step will eliminate all similar peptides of type II but is not guaranteed to remove all similar peptides of Type I. For example, this method will not eliminate one of the two peptides in Figure 5A although they share 84.6% sequence similarity.
- Step 2 filters the dataset using an 80% similarity threshold to eliminate any sequence that has a similarity of 80% or greater with one or more sequences in the dataset.

In addition, we also used a procedure proposed by Raghava [20] for similarity reduction of MHCBench benchmark datasets. Briefly, given two peptides *p*_1_ and *p*_2_ of lengths *l*_1_ and *l*_2_ such that *l*_1_≤*l*_2_, we compare *p*_1_ with each *l*_1_-length subpeptide in *p*_2_. If the percent identity (PID) between *p*_1_ and any subpeptide in *p*_2_ is greater than 80%, then the two peptides are deemed to be similar. For example, to compute the PID between (ACDEFGHIKLMNPQRST) and (DEFGGIKLMN), we compare (DEFGGIKLMN) with (ACDEFGHIKL), (CDEFGHIKLM), …, (IKLMNPQRST). The PID between (DEFGGIKLMN) and (DEFGHIKLMN) is 90% since nine out of 10 residues are identical.

Finally, we explored a method for assigning weights to similar peptides as opposed to eliminating similar peptides from the dataset. Specifically, the peptides within the binders category that are similar to each other (i.e., share a 9-mer subsequence or have sequence similarity of 80% or greater) are clustered together. Each peptide that is assigned to a cluster is similar to at least one other peptide within the cluster, and no two similar peptides are assigned to different clusters. Each peptide in a cluster is assigned a weight of 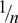, where *n* is the number of peptides assigned to the cluster. The process is repeated with peptides in the non-binders category. The result is a dataset of *weighted* instances.

Thus, from each MHC-II benchmark dataset, we generated five versions summarized below:

- Three datasets of *unique* peptides, MHCPEP-UPDS, MHCBN-UPDS, and IEDB-UPDS extracted from MHCPEP, MHCBN, and IEDB, respectively after eliminating short peptides consisting of fewer than 9 residues, unnatural peptides, peptides with greater than 75% Alanine residues, and duplicated peptides.
- Three datasets of *similarity-reduced* peptides, MHCPEP-SRDS1, MHCBN-SRDS1, and IEDB-SRDS1 derived from the corresponding UPDS datasets described above using only step 1 of the two-step similarity reduction procedure described above which ensures that no two peptides in the resulting datasets of binders or non binders share a 9-mer subsequence.
- Three datasets of *similarity-reduced* peptides, MHCPEP-SRDS2, MHCBN-SRDS2, and IEDB-SRDS2, extracted MHCPEP-SRDS1, MHCBN-SRDS1, and IEDB-SRDS1 respectively by filtering the binders and non-binders in SRDS1 such that the sequence identity between any pair of peptides in the binders category or in the non-binders category is less than 80%.
- Three datasets of *similarity-reduced* peptides, MHCPEP-SRDS3, MHCBN-SRDS3, and IEDB-SRDS3, derived from the corresponding UPDS datasets by applying the similarity reduction procedure introduced by Raghava which has been used to construct the MHCBench dataset [20].
- Three *weighted* unique peptide datasets, MHCPEP-WUPDS, MHCBN-WUPDS, and IEDB-WUPDS, derived from the corresponding UPDS datasets by applying the peptide weighting method described above.

**Figure 6:**
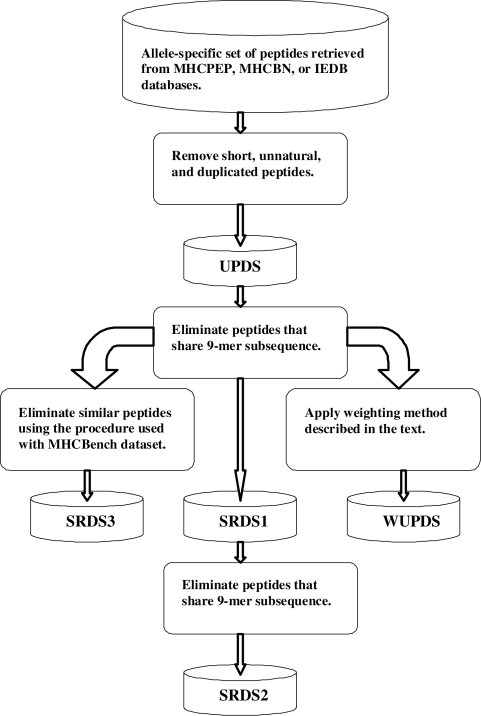
An overview of the process used for generating five different versions of each allele dataset using the different similarity-reduction methods described in the text.

The procedure used to generate the five different versions of each allele-specific dataset using the different similarity reduction methods and the peptide weighting method described above is shown in Figure 6. Note that UPDS can contain similar peptides of both types I and II; SRDS1 can contain similar peptides of type I; SRDS2 is free from both type I and type II similar peptides; SRDS3 simulates *similarity-reduced* datasets using the method employed with MHCBench; WUPDS is a *weighted* version of the UPDS dataset where similar peptides are grouped into disjoint clusters and the weight of each peptide is set to one over the size of its cluster.

### Summary of the datasets

#### Datasets derived from MHCPEP

Table 1 summarizes the number of binders in each *unique* peptides dataset, MHCPEP-UPDS, and the corresponding three *similarity-reduced* datasets, MHCPEP-SRDS1, MHCPEP-SRDS2, and MHCPEP-SRDS3. Note that on average, the number of binders in the *similarity-reduced* datasets, MHCPEP-SRDS1, MHCPEP-SRDS2, and MHCPEP-SRDS3, is reduced to 48%, 33%, and 39%, respectively, of the number of binders in MHCPEP-UPDS datasets.

#### Datasets derived from MHCBN

Table 2 summarizes the number of binders and non-binders in MHCBN-UPDS, MHCBN-SRDS1, MHCBN-SRDS2 and MHCBN-SRDS3 datasets derived for each of the eight MHCBN alleles satisfying our selection criteria. Note that the average number of binders in *similarity-reduced* datasets, MHCBN-SRDS1, MHCBN-SRDS2, and MHCBN-SRDS3, is reduced to 55.48%, 45.46%, and 61.39%, respectively, of the number of binders in MHCBN-UPDS datasets. Similarly, the average number of non-binders in *similarity-reduced* datasets, MHCBN-SRDS1, MHCBN-SRDS2, and MHCBN-SRDS3, is reduced to 67.55%, 64.24%, and 87.47%, respectively, of the number of non-binders in MHCBN-UPDS datasets.

#### Datasets derived from IEDB

Table 3 summarizes the number of binders and non-binders in IEDB-UPDS, IEDB-SRDS1, IEDB-SRDS2, and IEDB-SRDS3 datasets derived for 12 HLA alleles. We observed that the average number of binders in *similarity-reduced* datasets, IEDB-SRDS1, IEDB-SRDS2, and IEDB-SRDS3, is reduced to 51.17%, 47.66%, and 63.5%, respectively, of the number of binders in MHCBN-UPDS datasets. Similarly, the average number of non-binders in *similarity-reduced* datasets, IEDB-SRDS1, IEDB-SRDS2, and IEDB-SRDS3, is reduced to 60.86%, 59.38%, and 82.9%, respectively, of the number of non-binders in MHCBN-UPDS datasets.

### Independent blind set

Recently, Wang et al. [30] introduced a comprehensive dataset of previously unpublished MHC-II peptide binding affinities and utilized it to assessing the performance of nine publicly available MHC-II prediction methods. The dataset covers 14 HLA alleles and two Mouse alleles. Out of the 14 HLA allele-specific datasets, five datasets are used in our experiments as independent blind test data to evaluate the performance of the classifiers trained using the corresponding MHCBN allele-specific datasets. Table 19 shows the number of test peptides in each allele-specific dataset and the number of binders and non-binders obtained using an IC50 cutoff of 500 nM employed to categorize peptides into binders and non-binders [7].

**Table 19:** Five allele-specific blind test set obtained [30].

Allele	peptides	binders	non-binders
HLA-DRB1-0101	3882	2579	1303
HLA-DRB1-0301	502	209	293
HLA-DRB1-0401	512	286	226
HLA-DRB1-0701	505	358	147
HLA-DRB1-1101	520	317	203

Peptides are categorized into binders and non-binders using an IC50 cutoff 500 nM.

### Prediction methods

Our experiments focused on two approaches for training MHC-II binding peptide predictors from variable-length MHC-II peptides have been recently proposed in [16], [17] and a method based on *k*-spectrum kernel [24] that is designed to rely on the presence of high degree of sequence similarity between training and test peptides (and hence is expected to perform well on redundant datasets but poorly on *similarity-reduced* datasets). We implemented the three methods in java using Weka machine learning workbench [38]. Brief descriptions of each of the three prediction methods are included below.

### Composition-Transition-Distribution (CTD)

The basic idea of this approach is to map each variable-length peptide into a fixed-length feature vector such that standard machine learning algorithms are applicable. This method was used and explained in details in [16], [39]. 21 features are extracted from each peptide sequence as follows:

- First, each peptide sequence *p* is mapped into a string *s_p_* defined over an alphabet of three symbols, {1,2,3}. The mapping is performed by grouping amino acids into three groups using a physico-chemical property of amino acids (see Table 20). For example the peptide (AIRHIPRRIR) is mapped into (2312321131) using the hydrophobicity division of amino acids into three groups (see Table 20).
- Second, for each peptide string *s_p_*, three descriptors are derived as follows:
  - Composition (C): three features representing the percent frequency of the symbols, {1, 2, 3}, in the mapped peptide sequence.
  - Transition (T): three features representing the percent frequency of *i* followed by *j* or *j* followed by *i*, for *i*, *j*∈{1,2,3}.
  - Distribution (D): five features per symbol representing the fractions of the entire sequence where the first, 25, 50, 75, and 100% of the candidate symbol are contained in *s_p_*. A total of 15 features are derived from each peptide.

**Table 20:** Categorization of amino acids into three groups for a number of physicochemical properties [42].

Property	Group 1	Group 2	Group 3
Hydrophobicity	RKEDQN	GASTPHY	CVLIMFW
Polarizability	GASCTPD	NVEQIL	MHKFRYW
Polarity	LIFWCMVY	PATGS	HQRKNED
Van der Waal's volume	GASDT	CPNVEQIL	KMHFRYW

Table 20 shows division of the 20 amino acids into three groups based on hydrophobicity, polarizability, polarity, and Van der Waal's volume properties. Using these four properties, we derived 84 CTD features from each peptide sequence. In our experiments, we trained SVM classifiers using RBF kernel and peptide sequences represented using their amino acid sequence composition (20 features) and CTD descriptors (84 features).

### Local alignment (LA) kernel

Local alignment (LA) kernel [40] is a string kernel designed for biological sequence classification problems. The LA kernel measures the similarity between two sequences by adding up the scores obtained from local alignments with gaps of the sequences. This kernel has several parameters: the gap opening and extension penalty parameters *d* and *e*, the amino acid mutation matrix *s*, and the factor *β* which controls the influence of suboptimal alignments in the kernel value. Saigo et al. [40] used the BLOSUM62 substitution matrix, gap opening and extending parameters equal 11 and 1, respectively, and *β* ranges from 0.2 to 0.5. In our experiments, we tried a range of values for gap opening/extension and *β* parameters and got the best performance out of LA kernel using BLOSUM62 substitution matrix, gap opening and extending parameters equal 10 and 1, respectively, and *β* = 0.5. Detailed formulation of the LA kernel and a dynamic programming implementation of the kernel are provided in [40].

### *k*-spectrum kernel

Intuitively, a *k*-spectrum kernel [24] captures a simple notion of string similarity: two strings are deemed similar (i.e., have a high *k*-spectrum kernel value) if they share many of the same *k*-mer substrings. We used the *k*-spectrum with relatively large *k* value, *k* = 5. As noted earlier, the choice of a relatively large value for *k* was motivated by the desire to construct a predictor that is expected to perform well in settings where the peptides in the test set share significant similarity with one or more peptides in the training set.

### Performance evaluation

The prediction accuracy (ACC), sensitivity (Sn), specificity (Sp), and correlation coefficient (CC) are often used to evaluate prediction algorithms [26]. The CC measure has a value in the range from −1 to +1 and the closer the value to +1, the better the predictor. The Sn and Sp summarize the accuracies of the positive and negative predictions respectively. ACC, Sn, Sp, and CC are defined as follows:
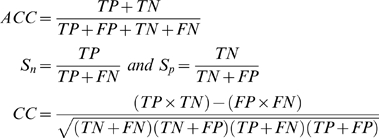
where *TP*, *FP*, *TN*, *and FN* are the numbers of true positives, false positives, true negatives, and false negatives respectively.

Although these metrics are widely used to assess the performance of machine learning methods, they all suffer from an important limitation of being threshold-dependent. Threshold-dependent metrics describe the classifier performance at a specific threshold value. It is often possible to increase the number of true positives (equivalently, the sensitivity) of the classifier at the expense of an increase in false positives (equivalently, the false alarm rate). The ROC (Receiver Operating Characteristic) curve shows the performance of the classifier over all possible thresholds. The ROC curve is obtained by plotting the true positive rate as a function of the false positive rate or, equivalently, sensitivity versus (1-specificity) as the discrimination threshold of the binary classifier is varied. Each point on the ROC curve describes the classifier at a certain threshold value and, hence, a particular choice of tradeoff between true positive rate and false negative rate. The area under ROC curve (AUC) is a useful summary statistic for comparing two ROC curves. The AUC is defined as the probability that a randomly chosen positive example will be ranked higher than a randomly chosen negative example. An ideal classifier will have an AUC = 1, while a classifier performs no better than random will have an AUC = 0.5, any classifier performing better than random will have an AUC value that lies between these two extremes.

### Implementation and SVM parameter optimization

We used the Weka machine learning workbench [38] for implementing the spectrum, and LA kernels (RBF kernel is already implemented in Weka). For the SVM classifier, we used the weka implementation of the SMO algorithm [41]. For *k*-spectrum and LA kernels, the default value of the cost parameter, *C* = 1, was used for the SMO classifier. For the RBF kernel, we found that tuning the SMO cost parameter *C* and the RBF kernel parameter *γ* is necessary to obtain satisfactory performance. We tuned these parameters using a two dimensional grid search over the range *C* = 2^−5^,2^−3^,…,2^3^, *γ* = 2^−15^,2^−13^,…,2^3^.

## Supporting Information

Data S1 Detailed results on MHCPEP, MHCBN, and IEDB datasets
(0.12 MB PDF)

Dataset S1 Datasets derived from MHCPEP database
(0.36 MB ZIP)

Dataset S2 Datasets derived from MHCBN database
(0.17 MB ZIP)

Dataset S3 Datasets derived from IEDB database
(0.26 MB ZIP)

Code S1 Java programs implementing the similarity-reduction and peptide weighting methods
(0.01 MB ZIP)
