# Supplementary material for: On Evaluating MHC-II Binding Peptide Prediction Methods
Source: PLoS One. 2008 Sep 24;3(9):e3268. doi: 10.1371/journal.pone.0003268 (PMC2533399; doi:10.1371/journal.pone.0003268)
Supplement: Data S1 — Detailed results on MHCPEP, MHCBN, and IEDB datasets (0.12 MB PDF) [file pone.0003268.s001.pdf]

Supplementary Materials
On Evaluating MHC-II Binding Peptide Prediction Methods

Table S1: Performance of CTD method on MHCPEP-UPDS datasets using 5-fold cross-validation test. Last column refers to the number of binding peptides. For each dataset an equal number of non-binding peptide is generated randomly form Swissprot sequences (see Methods section for details).

allele	ACC	Sn	Sp	CC	AUC	N
HLA-DQ2	88.05	81.42	94.69	0.768	0.939	113
HLA-DQ4	86.60	91.75	81.44	0.736	0.934	97
HLA-DQ7	80.00	80.00	80.00	0.600	0.853	135
HLA-DR1	81.29	76.10	86.49	0.629	0.863	703
HLA-DR2	79.05	72.38	85.71	0.586	0.829	315
HLA-DR3	83.85	73.96	93.75	0.691	0.862	192
HLA-DR4	86.59	73.36	99.82	0.759	0.906	1085
HLA-DR5	82.01	64.02	100.00	0.686	0.887	189
HLA-DR7	87.10	80.35	93.84	0.749	0.904	341
HLA-DR8	86.00	79.20	92.80	0.727	0.903	125
HLA-DR9	86.17	75.53	96.81	0.740	0.913	94
HLA-DR11	91.65	90.70	92.60	0.833	0.958	473
HLA-DR13	87.60	77.69	97.52	0.767	0.920	121
HLA-DR15	83.06	73.55	92.56	0.673	0.867	121
HLA-DR17	90.82	86.08	95.57	0.820	0.934	158
HLA-DR51	83.91	75.65	92.17	0.688	0.886	115
I-Ab	81.99	72.79	91.18	0.651	0.875	136
I-Ad	86.27	79.52	93.01	0.732	0.902	415
I-Ag7	82.80	76.43	89.17	0.661	0.887	157
I-Ak	83.66	76.77	90.55	0.680	0.881	254
I-Ed	88.61	83.33	93.88	0.776	0.936	294
I-Ek	89.82	84.13	95.51	0.802	0.951	334

Table S2: Performance of CTD method on MHCPEP-SRDS1 datasets using 5-fold cross-validation test. Last column refers to the number of binding peptides. For each dataset an equal number of non-binding peptide is generated randomly form Swissprot sequences (see Methods section for details).

allele	ACC	Sn	Sp	CC	AUC	N
HLA-DQ2	82.09	79.10	85.07	0.643	0.853	67
HLA-DQ4	81.55	80.95	82.14	0.631	0.881	84
HLA-DQ7	69.86	61.64	78.08	0.403	0.726	73
HLA-DR1	70.83	64.58	77.08	0.420	0.744	336
HLA-DR2	71.96	69.59	74.32	0.440	0.781	148
HLA-DR3	59.88	19.75	100.00	0.331	0.572	81
HLA-DR4	69.93	66.06	73.80	0.400	0.723	439
HLA-DR5	71.20	65.22	77.17	0.427	0.784	92
HLA-DR7	69.34	66.42	72.26	0.388	0.732	137
HLA-DR8	68.09	68.09	68.09	0.362	0.694	47
HLA-DR9	71.95	68.29	75.61	0.440	0.721	41
HLA-DR11	83.13	79.38	86.88	0.664	0.890	160
HLA-DR13	78.68	79.41	77.94	0.574	0.837	68
HLA-DR15	66.67	68.75	64.58	0.334	0.667	48
HLA-DR17	82.32	71.95	92.68	0.661	0.876	82
HLA-DR51	61.11	44.44	77.78	0.236	0.603	45
I-Ab	62.10	62.90	61.29	0.242	0.680	62
I-Ad	75.30	76.19	74.40	0.506	0.804	168
I-Ag7	66.94	58.06	75.81	0.344	0.702	62
I-Ak	71.35	72.92	69.79	0.427	0.796	96
I-Ed	90.16	84.57	95.74	0.808	0.932	188
I-Ek	86.76	87.25	86.27	0.735	0.941	204

Table S3: Performance of CTD method on MHCPEP-SRDS2 datasets using 5-fold cross-validation test. Last column refers to the number of binding peptides. For each dataset an equal number of non-binding peptide is generated randomly form Swissprot sequences (see Methods section for details).

allele	ACC	Sn	Sp	CC	AUC	N
HLA-DQ2	64.06	53.13	75.00	0.288	0.573	32
HLA-DQ4	86.08	86.08	86.08	0.722	0.817	79
HLA-DQ7	66.15	67.69	64.62	0.323	0.709	65
HLA-DR1	64.67	64.46	64.88	0.293	0.711	242
HLA-DR2	60.58	57.69	63.46	0.212	0.614	104
HLA-DR3	57.97	55.07	60.87	0.160	0.572	69
HLA-DR4	64.77	60.40	69.13	0.296	0.692	298
HLA-DR5	64.75	70.49	59.02	0.297	0.625	61
HLA-DR7	57.47	57.47	57.47	0.149	0.599	87
HLA-DR8	63.04	60.87	65.22	0.261	0.680	46
HLA-DR9	67.65	55.88	79.41	0.363	0.690	34
HLA-DR11	74.00	75.00	73.00	0.480	0.792	100
HLA-DR13	60.29	58.82	61.76	0.206	0.587	34
HLA-DR15	68.06	72.22	63.89	0.362	0.689	36
HLA-DR17	53.75	50.00	57.50	0.075	0.550	40
HLA-DR51	60.26	66.67	53.85	0.207	0.595	39
I-Ab	62.75	62.75	62.75	0.255	0.638	51
I-Ad	57.92	58.42	57.43	0.158	0.618	101
I-Ag7	66.98	64.15	69.81	0.340	0.632	53
I-Ak	63.43	62.69	64.18	0.269	0.661	67
I-Ed	68.75	71.43	66.07	0.376	0.744	56
I-Ek	68.75	70.31	67.19	0.375	0.703	64

Table S4: Performance of CTD method on MHCPEP-SRDS3 datasets using 5-fold cross-validation test. Last column refers to the number of binding peptides. For each dataset an equal number of non-binding peptide is generated randomly form Swissprot sequences (see Methods section for details).

allele	ACC	Sn	Sp	CC	AUC	N
HLA-DQ2	75.64	71.79	79.49	0.514	0.754	39
HLA-DQ4	84.15	90.24	78.05	0.688	0.900	82
HLA-DQ7	64.67	60.00	69.33	0.295	0.706	75
HLA-DR1	66.37	67.63	65.11	0.327	0.720	278
HLA-DR2	64.18	59.70	68.66	0.285	0.686	134
HLA-DR3	56.16	50.68	61.64	0.124	0.532	73
HLA-DR4	65.01	61.47	68.56	0.301	0.695	353
HLA-DR5	60.00	64.00	56.00	0.201	0.617	75
HLA-DR7	60.40	59.41	61.39	0.208	0.613	101
HLA-DR8	66.67	62.96	70.37	0.334	0.714	54
HLA-DR9	74.32	75.68	72.97	0.487	0.792	37
HLA-DR11	79.61	79.61	79.61	0.592	0.850	103
HLA-DR13	61.11	63.89	58.33	0.223	0.630	36
HLA-DR15	66.33	65.31	67.35	0.327	0.663	49
HLA-DR17	67.78	71.11	64.44	0.356	0.682	45
HLA-DR51	64.55	67.27	61.82	0.291	0.672	55
I-Ab	61.48	54.10	68.85	0.232	0.618	61
I-Ad	65.56	65.93	65.19	0.311	0.676	135
I-Ag7	69.75	58.02	81.48	0.406	0.757	81
I-Ak	65.29	62.35	68.24	0.306	0.691	85
I-Ed	73.03	73.68	72.37	0.461	0.787	76
I-Ek	70.51	65.38	75.64	0.412	0.699	78

Table S5: Performance of CTD method on MHCPEP-WUPDS datasets using 5-fold cross-validation test. Each dataset is obtained by applying the peptide weighting procedure separately to the binders and non-binders in the corresponding MHCPEP-UPDS dataset (see Methods section for details).

allele	ACC	Sn	Sp	CC	AUC
HLA-DQ2	85.65	33.32	100.00	0.531	0.772
HLA-DQ4	86.00	87.80	84.54	0.720	0.925
HLA-DQ7	76.74	71.51	79.26	0.492	0.812
HLA-DR1	80.98	36.61	93.60	0.372	0.699
HLA-DR2	77.86	12.67	100.00	0.313	0.617
HLA-DR3	77.55	17.67	100.00	0.367	0.614
HLA-DR4	85.41	31.98	99.91	0.516	0.730
HLA-DR5	80.16	25.32	100.00	0.447	0.733
HLA-DR7	84.54	23.92	100.00	0.448	0.632
HLA-DR8	87.67	53.42	100.00	0.676	0.802
HLA-DR9	95.67	44.75	100.00	0.654	0.738
HLA-DR11	89.47	54.88	96.82	0.600	0.830
HLA-DR13	82.49	20.71	100.00	0.411	0.605
HLA-DR15	80.02	19.58	100.00	0.393	0.587
HLA-DR17	88.17	42.56	100.00	0.609	0.679
HLA-DR51	82.95	35.12	100.00	0.534	0.718
I-Ab	78.59	25.41	98.53	0.393	0.636
I-Ad	85.60	37.62	98.31	0.506	0.707
I-Ag7	80.93	26.51	100.00	0.459	0.700
I-Ak	84.18	23.29	100.00	0.441	0.680
I-Ed	88.93	17.18	97.31	0.221	0.805
I-Ek	88.93	17.18	97.31	0.221	0.805

Table S6: Performance of LA method on MHCPEP-UPDS datasets using 5-fold cross-validation test. Last column refers to the number of binding peptides. For each dataset an equal number of non-binding peptide is generated randomly form Swissprot sequences (see Methods section for details).

allele	ACC	Sn	Sp	CC	AUC	N
HLA-DQ2	82.30	80.53	84.07	0.646	0.905	113
HLA-DQ4	79.38	74.23	84.54	0.591	0.903	97
HLA-DQ7	75.19	74.07	76.30	0.504	0.860	135
HLA-DR1	80.30	73.68	86.91	0.611	0.872	703
HLA-DR2	78.41	73.02	83.81	0.572	0.866	315
HLA-DR3	77.86	75.52	80.21	0.558	0.869	192
HLA-DR4	77.10	71.71	82.49	0.545	0.862	1085
HLA-DR5	80.16	75.13	85.19	0.606	0.864	189
HLA-DR7	79.18	72.73	85.63	0.588	0.858	341
HLA-DR8	83.60	80.80	86.40	0.673	0.896	125
HLA-DR9	79.79	77.66	81.91	0.596	0.880	94
HLA-DR11	86.68	80.34	93.02	0.740	0.938	473
HLA-DR13	80.99	74.38	87.60	0.625	0.905	121
HLA-DR15	77.69	74.38	80.99	0.555	0.829	121
HLA-DR17	83.86	79.75	87.97	0.680	0.907	158
HLA-DR51	80.87	79.13	82.61	0.618	0.891	115
I-Ab	78.68	77.21	80.15	0.574	0.855	136
I-Ad	81.81	75.42	88.19	0.641	0.898	415
I-Ag7	79.94	77.71	82.17	0.599	0.896	157
I-Ak	80.12	76.38	83.86	0.604	0.872	254
I-Ed	83.84	77.21	90.48	0.683	0.921	294
I-Ek	86.53	81.74	91.32	0.734	0.940	334

Table S7: Performance of LA method on MHCPEP-SRDS1 datasets using 5-fold cross-validation test. Last column refers to the number of binding peptides. For each dataset an equal number of non-binding peptide is generated randomly form Swissprot sequences (see Methods section for details).

allele	ACC	Sn	Sp	CC	AUC	N
HLA-DQ2	73.88	76.12	71.64	0.478	0.852	67
HLA-DQ4	78.57	72.62	84.52	0.576	0.854	84
HLA-DQ7	71.23	68.49	73.97	0.425	0.799	73
HLA-DR1	72.02	66.37	77.68	0.443	0.801	336
HLA-DR2	71.28	70.95	71.62	0.426	0.795	148
HLA-DR3	60.49	61.73	59.26	0.210	0.678	81
HLA-DR4	68.56	63.78	73.35	0.373	0.751	439
HLA-DR5	70.65	70.65	70.65	0.413	0.776	92
HLA-DR7	62.77	62.77	62.77	0.255	0.702	137
HLA-DR8	57.45	38.30	76.60	0.161	0.625	47
HLA-DR9	63.41	75.61	51.22	0.277	0.746	41
HLA-DR11	82.81	80.63	85.00	0.657	0.912	160
HLA-DR13	72.06	75.00	69.12	0.442	0.827	68
HLA-DR15	62.50	66.67	58.33	0.251	0.718	48
HLA-DR17	72.56	74.39	70.73	0.452	0.806	82
HLA-DR51	65.56	75.56	55.56	0.318	0.788	45
I-Ab	66.94	54.84	79.03	0.349	0.705	62
I-Ad	72.32	69.64	75.00	0.447	0.818	168
I-Ag7	74.19	70.97	77.42	0.485	0.778	62
I-Ak	75.00	76.04	73.96	0.500	0.800	96
I-Ed	82.45	77.13	87.77	0.653	0.903	188
I-Ek	84.31	78.43	90.20	0.691	0.913	204

Table S8: Performance of LA method on MHCPEP-SRDS2 datasets using 5-fold cross-validation test. Last column refers to the number of binding peptides. For each dataset an equal number of non-binding peptide is generated randomly form Swissprot sequences (see Methods section for details).

allele	ACC	Sn	Sp	CC	AUC	N
HLA-DQ2	65.63	71.88	59.38	0.315	0.678	32
HLA-DQ4	88.61	83.54	93.67	0.776	0.954	79
HLA-DQ7	67.69	70.77	64.62	0.355	0.713	65
HLA-DR1	64.46	61.16	67.77	0.290	0.715	242
HLA-DR2	61.54	65.38	57.69	0.231	0.633	104
HLA-DR3	55.80	63.77	47.83	0.117	0.602	69
HLA-DR4	64.43	65.10	63.76	0.289	0.711	298
HLA-DR5	53.28	63.93	42.62	0.067	0.622	61
HLA-DR7	60.92	66.67	55.17	0.220	0.622	87
HLA-DR8	64.13	71.74	56.52	0.286	0.717	46
HLA-DR9	70.59	64.71	76.47	0.415	0.754	34
HLA-DR11	73.50	69.00	78.00	0.472	0.810	100
HLA-DR13	73.53	76.47	70.59	0.471	0.827	34
HLA-DR15	58.33	58.33	58.33	0.167	0.698	36
HLA-DR17	57.50	62.50	52.50	0.151	0.612	40
HLA-DR51	64.10	71.79	56.41	0.285	0.664	39
I-Ab	60.78	56.86	64.71	0.216	0.624	51
I-Ad	60.40	56.44	64.36	0.209	0.700	101
I-Ag7	70.75	64.15	77.36	0.419	0.756	53
I-Ak	63.43	55.22	71.64	0.272	0.664	67
I-Ed	69.64	71.43	67.86	0.393	0.760	56
I-Ek	64.06	64.06	64.06	0.281	0.756	64

Table S9: Performance of LA method on MHCPEP-SRDS3 datasets using 5-fold cross-validation test. Last column refers to the number of binding peptides. For each dataset an equal number of non-binding peptide is generated randomly form Swissprot sequences (see Methods section for details).

allele	ACC	Sn	Sp	CC	AUC	N
HLA-DQ2	65.38	58.97	71.79	0.310	0.655	39
HLA-DQ4	79.88	74.39	85.37	0.601	0.900	82
HLA-DQ7	70.67	72.00	69.33	0.413	0.757	75
HLA-DR1	66.37	66.91	65.83	0.327	0.747	278
HLA-DR2	64.18	67.16	61.19	0.284	0.736	134
HLA-DR3	64.38	67.12	61.64	0.288	0.657	73
HLA-DR4	64.02	62.89	65.16	0.281	0.717	353
HLA-DR5	64.00	73.33	54.67	0.285	0.723	75
HLA-DR7	67.82	74.26	61.39	0.359	0.765	101
HLA-DR8	69.44	70.37	68.52	0.389	0.732	54
HLA-DR9	72.97	72.97	72.97	0.459	0.779	37
HLA-DR11	78.16	74.76	81.55	0.564	0.854	103
HLA-DR13	63.89	69.44	58.33	0.280	0.623	36
HLA-DR15	59.18	67.35	51.02	0.186	0.713	49
HLA-DR17	68.89	60.00	77.78	0.384	0.769	45
HLA-DR51	65.45	72.73	58.18	0.312	0.780	55
I-Ab	61.48	50.82	72.13	0.235	0.611	61
I-Ad	67.41	70.37	64.44	0.349	0.785	135
I-Ag7	72.22	76.54	67.90	0.446	0.804	81
I-Ak	71.18	65.88	76.47	0.426	0.766	85
I-Ed	78.95	84.21	73.68	0.582	0.852	76
I-Ek	66.67	71.79	61.54	0.335	0.789	78

Table S10: Performance of LA method on MHCPEP-WUPDS datasets using 5-fold cross-validation test. Each dataset is obtained by applying the peptide weighting procedure separately to the binders and non-binders in the corresponding MHCPEP-UPDS dataset (see Methods section for details).

allele	ACC	Sn	Sp	CC	AUC
HLA-DQ2	77.24	42.68	86.73	0.304	0.738
HLA-DQ4	77.27	68.35	84.54	0.539	0.882
HLA-DQ7	73.67	54.35	82.96	0.385	0.786
HLA-DR1	79.57	28.27	94.17	0.300	0.710
HLA-DR2	75.01	30.41	90.16	0.250	0.688
HLA-DR3	71.32	39.73	83.33	0.245	0.678
HLA-DR4	79.38	22.50	94.81	0.249	0.723
HLA-DR5	79.30	42.65	92.55	0.416	0.709
HLA-DR7	78.78	12.85	95.60	0.142	0.599
HLA-DR8	82.21	66.11	88.00	0.542	0.810
HLA-DR9	93.73	57.50	96.81	0.556	0.819
HLA-DR11	83.84	34.20	94.37	0.352	0.798
HLA-DR13	75.32	23.53	90.00	0.167	0.714
HLA-DR15	75.78	40.00	87.60	0.303	0.627
HLA-DR17	82.91	39.02	94.30	0.407	0.760
HLA-DR51	74.89	53.24	82.61	0.356	0.786
I-Ab	74.06	46.08	84.56	0.320	0.669
I-Ad	82.49	32.81	95.66	0.381	0.740
I-Ag7	78.40	57.14	85.99	0.436	0.797
I-Ak	79.33	33.09	91.34	0.287	0.684
I-Ed	95.46	24.00	98.63	0.303	0.760
I-Ek	90.83	30.28	97.90	0.394	0.824

Table S11: Performance of SVM using 5-spectrum kernel method on MHCPEP-UPDS datasets using 5-fold cross-validation test. Last column refers to the number of binding peptides. For each dataset an equal number of non-binding peptide is generated randomly form Swissprot sequences (see Methods section for details).

allele	ACC	Sn	Sp	CC	AUC	N
HLA-DQ2	89.38	78.76	100.00	0.806	0.908	113
HLA-DQ4	63.40	26.80	100.00	0.393	0.628	97
HLA-DQ7	84.44	68.89	100.00	0.725	0.856	135
HLA-DR1	86.13	72.26	100.00	0.752	0.883	703
HLA-DR2	86.19	72.38	100.00	0.753	0.884	315
HLA-DR3	86.46	72.92	100.00	0.757	0.854	192
HLA-DR4	90.55	81.29	99.82	0.825	0.917	1085
HLA-DR5	88.89	77.78	100.00	0.798	0.905	189
HLA-DR7	91.35	82.70	100.00	0.840	0.916	341
HLA-DR8	89.60	79.20	100.00	0.810	0.894	125
HLA-DR9	85.11	70.21	100.00	0.736	0.836	94
HLA-DR11	90.27	80.55	100.00	0.821	0.910	473
HLA-DR13	88.02	76.03	100.00	0.783	0.875	121
HLA-DR15	85.54	71.07	100.00	0.742	0.887	121
HLA-DR17	91.46	82.91	100.00	0.841	0.907	158
HLA-DR51	87.83	75.65	100.00	0.780	0.924	115
I-Ab	87.87	75.74	100.00	0.781	0.865	136
I-Ad	91.93	83.86	100.00	0.850	0.942	415
I-Ag7	89.49	78.98	100.00	0.808	0.916	157
I-Ak	88.39	76.77	100.00	0.789	0.909	254
I-Ed	89.29	78.57	100.00	0.804	0.918	294
I-Ek	90.42	80.84	100.00	0.824	0.934	334

Table S12: Performance of SVM using 5-spectrum kernel method on MHCPEP-SRDS1 datasets using 5-fold cross-validation test. Last column refers to the number of binding peptides. For each dataset an equal number of non-binding peptide is generated randomly form Swissprot sequences (see Methods section for details).

allele	ACC	Sn	Sp	CC	AUC	N
HLA-DQ2	78.36	56.72	100.00	0.629	0.789	67
HLA-DQ4	57.14	15.48	98.81	0.258	0.544	84
HLA-DQ7	65.07	31.51	98.63	0.407	0.677	73
HLA-DR1	67.56	36.01	99.11	0.453	0.662	336
HLA-DR2	68.58	37.16	100.00	0.478	0.694	148
HLA-DR3	62.35	24.69	100.00	0.375	0.603	81
HLA-DR4	70.96	43.28	98.63	0.503	0.710	439
HLA-DR5	72.83	45.65	100.00	0.544	0.691	92
HLA-DR7	70.80	42.34	99.27	0.506	0.721	137
HLA-DR8	54.26	8.51	100.00	0.211	0.552	47
HLA-DR9	63.41	26.83	100.00	0.394	0.620	41
HLA-DR11	70.31	40.63	100.00	0.505	0.703	160
HLA-DR13	77.21	54.41	100.00	0.611	0.746	68
HLA-DR15	69.79	39.58	100.00	0.497	0.711	48
HLA-DR17	80.49	60.98	100.00	0.662	0.789	82
HLA-DR51	66.67	33.33	100.00	0.447	0.651	45
I-Ab	64.52	29.03	100.00	0.412	0.620	62
I-Ad	78.27	56.55	100.00	0.628	0.787	168
I-Ag7	72.58	45.16	100.00	0.540	0.718	62
I-Ak	72.92	45.83	100.00	0.545	0.761	96
I-Ed	81.12	62.23	100.00	0.672	0.826	188
I-Ek	85.29	70.59	100.00	0.739	0.874	204

Table S13: Performance of SVM using 5-spectrum kernel method on MHCPEP-SRDS2 datasets using 5-fold cross-validation test. Last column refers to the number of binding peptides. For each dataset an equal number of non-binding peptide is generated randomly form Swissprot sequences (see Methods section for details).

allele	ACC	Sn	Sp	CC	AUC	N
HLA-DQ2	53.13	28.13	78.13	0.072	0.566	32
HLA-DQ4	57.59	16.46	98.73	0.267	0.590	79
HLA-DQ7	61.54	24.62	98.46	0.342	0.616	65
HLA-DR1	55.58	12.81	98.35	0.215	0.562	242
HLA-DR2	54.81	30.77	78.85	0.110	0.548	104
HLA-DR3	51.45	24.64	78.26	0.034	0.514	69
HLA-DR4	56.04	33.22	78.86	0.136	0.578	298
HLA-DR5	58.20	16.39	100.00	0.299	0.583	61
HLA-DR7	55.17	31.03	79.31	0.118	0.562	87
HLA-DR8	51.09	21.74	80.43	0.027	0.526	46
HLA-DR9	48.53	58.82	38.24	-0.030	0.488	34
HLA-DR11	51.50	43.00	60.00	0.030	0.528	100
HLA-DR13	50.00	41.18	58.82	0.000	0.518	34
HLA-DR15	59.72	38.89	80.56	0.214	0.592	36
HLA-DR17	56.25	32.50	80.00	0.142	0.568	40
HLA-DR51	60.26	20.51	100.00	0.338	0.578	39
I-Ab	54.90	49.02	60.78	0.099	0.570	51
I-Ad	62.87	27.72	98.02	0.362	0.623	101
I-Ag7	70.75	41.51	100.00	0.512	0.713	53
I-Ak	61.19	23.88	98.51	0.336	0.586	67
I-Ed	66.07	32.14	100.00	0.438	0.645	56
I-Ek	60.16	39.06	81.25	0.224	0.606	64

Table S14: Performance of SVM using 5-spectrum kernel method on MHCPEP-SRDS3 datasets using 5-fold cross-validation test. Last column refers to the number of binding peptides. For each dataset an equal number of non-binding peptide is generated randomly form Swissprot sequences (see Methods section for details).

allele	ACC	Sn	Sp	CC	AUC	N
HLA-DQ2	70.51	41.03	100.00	0.508	0.663	39
HLA-DQ4	59.15	18.29	100.00	0.317	0.608	82
HLA-DQ7	69.33	38.67	100.00	0.490	0.699	75
HLA-DR1	66.73	34.17	99.28	0.441	0.676	278
HLA-DR2	73.13	46.27	100.00	0.549	0.724	134
HLA-DR3	58.22	16.44	100.00	0.299	0.623	73
HLA-DR4	68.56	38.81	98.30	0.462	0.679	353
HLA-DR5	72.00	45.33	98.67	0.520	0.719	75
HLA-DR7	64.85	30.69	99.01	0.407	0.631	101
HLA-DR8	64.81	29.63	100.00	0.417	0.608	54
HLA-DR9	51.35	24.32	78.38	0.032	0.520	37
HLA-DR11	52.43	44.66	60.19	0.049	0.544	103
HLA-DR13	55.56	30.56	80.56	0.128	0.563	36
HLA-DR15	77.55	55.10	100.00	0.617	0.805	49
HLA-DR17	64.44	28.89	100.00	0.411	0.629	45
HLA-DR51	82.73	65.45	100.00	0.697	0.800	55
I-Ab	67.21	34.43	100.00	0.456	0.606	61
I-Ad	81.48	62.96	100.00	0.678	0.821	135
I-Ag7	79.63	59.26	100.00	0.649	0.823	81
I-Ak	74.71	49.41	100.00	0.573	0.768	85
I-Ed	79.61	59.21	100.00	0.649	0.828	76
I-Ek	73.08	46.15	100.00	0.548	0.714	78

Table S15: Performance of SVM using 5-spectrum kernel method on MHCPEP-WUPDS datasets using 5-fold cross-validation test. Each dataset is obtained by applying the peptide weighting procedure separately to the binders and non-binders in the corresponding MHCPEP-UPDS dataset (see Methods section for details).

allele	ACC	Sn	Sp	CC	AUC
HLA-DQ2	35.38	90.13	20.35	0.112	0.717
HLA-DQ4	59.66	10.13	100.00	0.242	0.543
HLA-DQ7	80.50	40.00	100.00	0.557	0.716
HLA-DR1	23.37	100.00	1.56	0.059	0.696
HLA-DR2	82.03	29.63	100.00	0.489	0.682
HLA-DR3	80.11	27.08	100.00	0.461	0.612
HLA-DR4	87.14	42.14	99.35	0.580	0.723
HLA-DR5	85.16	44.12	100.00	0.606	0.765
HLA-DR7	88.08	41.38	100.00	0.600	0.714
HLA-DR8	90.00	62.22	100.00	0.740	0.796
HLA-DR9	92.16	NaN	92.16	NaN	0.806
HLA-DR11	85.57	17.60	100.00	0.387	0.612
HLA-DR13	83.64	25.91	100.00	0.463	0.620
HLA-DR15	84.01	35.63	100.00	0.542	0.760
HLA-DR17	75.88	60.98	79.75	0.364	0.747
HLA-DR51	87.29	51.63	100.00	0.664	0.838
I-Ab	82.89	37.25	100.00	0.549	0.650
I-Ad	90.15	52.99	100.00	0.686	0.815
I-Ag7	88.68	56.36	100.00	0.699	0.820
I-Ak	89.26	47.92	100.00	0.650	0.778
I-Ed	97.56	42.62	100.00	0.645	0.742
I-Ek	78.25	61.23	80.24	0.297	0.734

Table S16: Performance of CTD method on MHCBN-UPDS datasets using 5-fold cross-validation test.

allele	ACC	Sn	Sp	CC	AUC	binders	Non-binders
HLA-DR1	79.90	91.51	38.89	0.351	0.789	636	180
HLA-DR2	80.14	93.51	47.02	0.476	0.786	416	168
HLA-DR5	72.12	79.36	63.01	0.431	0.752	218	173
HLA-DRB10101	83.59	91.71	49.61	0.442	0.804	531	127
HLA-DRB10301	72.51	73.95	70.87	0.448	0.787	261	230
HLA-DRB10401	82.31	96.15	26.87	0.331	0.721	805	201
HLA-DRB10701	78.20	91.44	42.06	0.390	0.732	292	107
HLA-DRB11101	79.14	85.23	63.50	0.485	0.832	352	137

Table S17: Performance of CTD method on MHCBN-SRDS1 datasets using 5-fold cross-validation test.

allele	ACC	Sn	Sp	CC	AUC	binders	Non-binders
HLA-DR1	79.73	93.90	37.84	0.396	0.738	328	111
HLA-DR2	73.52	87.82	50.81	0.423	0.750	197	124
HLA-DR5	64.88	55.86	72.52	0.288	0.692	111	131
HLA-DRB10101	81.60	93.54	37.50	0.377	0.748	325	88
HLA-DRB10301	63.41	64.23	62.67	0.269	0.661	137	150
HLA-DRB10401	77.76	92.36	27.21	0.251	0.655	471	136
HLA-DRB10701	74.49	86.59	42.65	0.318	0.748	179	68
HLA-DRB11101	75.00	89.20	40.23	0.338	0.759	213	87

Table S18: Performance of CTD method on MHCBN-SRDS2 datasets using 5-fold cross-validation test.

allele	ACC	Sn	Sp	CC	AUC	binders	Non-binders
HLA-DR1	72.04	88.79	36.79	0.302	0.698	223	106
HLA-DR2	68.84	78.43	56.91	0.363	0.716	153	123
HLA-DR5	56.94	31.25	72.87	0.044	0.588	80	129
HLA-DRB10101	80.56	96.06	23.68	0.296	0.683	279	76
HLA-DRB10301	59.93	59.06	60.69	0.197	0.628	127	145
HLA-DRB10401	75.72	92.08	20.17	0.166	0.620	404	119
HLA-DRB10701	70.64	87.50	31.82	0.229	0.696	152	66
HLA-DRB11101	74.35	83.52	55.17	0.399	0.757	182	87

Table S19: Performance of CTD method on MHCBN-SRDS3 datasets using 5-fold cross-validation test.

allele	ACC	Sn	Sp	CC	AUC	binders	Non-binders
HLA-DR1	73.78	86.49	48.46	0.380	0.743	259	130
HLA-DR2	73.01	87.26	44.62	0.355	0.719	259	130
HLA-DR5	58.66	40.00	70.78	0.112	0.608	100	154
HLA-DRB10101	81.67	95.13	34.82	0.390	0.725	390	112
HLA-DRB10301	64.62	60.57	67.91	0.285	0.675	175	215
HLA-DRB10401	77.68	95.21	22.99	0.270	0.710	543	174
HLA-DRB10701	71.15	87.79	32.61	0.242	0.671	213	92
HLA-DRB11101	74.59	82.01	61.07	0.437	0.775	239	131

Table S20: Performance of CTD method on MHCBN-WUPDS datasets using 5-fold cross-validation test.

allele	ACC	Sn	Sp	CC	AUC
HLA-DR1	72.93	85.40	47.30	0.353	0.732
HLA-DR2	68.90	79.62	55.88	0.367	0.740
HLA-DR5	61.84	48.95	69.89	0.189	0.543
HLA-DRB10101	76.62	87.32	41.68	0.310	0.666
HLA-DRB10301	65.27	61.04	68.88	0.300	0.678
HLA-DRB10401	81.36	94.44	15.97	0.149	0.701
HLA-DRB10701	71.36	86.44	35.55	0.251	0.702
HLA-DRB11101	73.57	82.11	56.28	0.392	0.789

Table S21: Performance of LA method on MHCBN-UPDS datasets using 5-fold cross-validation test.

allele	ACC	Sn	Sp	CC	AUC	binders	Non-binders
HLA-DR1	80.27	95.75	25.56	0.310	0.768	636	180
HLA-DR2	77.91	95.67	33.93	0.401	0.771	416	168
HLA-DR5	69.57	62.84	78.03	0.408	0.748	218	173
HLA-DRB10101	81.76	96.80	18.90	0.256	0.799	531	127
HLA-DRB10301	71.89	73.56	70.00	0.436	0.795	261	230
HLA-DRB10401	82.80	95.53	31.84	0.366	0.780	805	201
HLA-DRB10701	82.46	93.84	51.40	0.518	0.842	292	107
HLA-DRB11101	80.16	90.34	54.01	0.480	0.874	352	137

Table S22: Performance of LA method on MHCBN-SRDS1 datasets using 5-fold cross-validation test.

allele	ACC	Sn	Sp	CC	AUC	binders	Non-binders
HLA-DR1	79.04	93.60	36.04	0.372	0.784	328	111
HLA-DR2	67.29	78.68	49.19	0.291	0.707	197	124
HLA-DR5	64.88	35.14	90.08	0.306	0.657	111	131
HLA-DRB10101	78.21	95.08	15.91	0.173	0.690	325	88
HLA-DRB10301	58.54	59.85	57.33	0.172	0.617	137	150
HLA-DRB10401	76.94	95.75	11.76	0.133	0.676	471	136
HLA-DRB10701	71.26	88.27	26.47	0.181	0.665	179	68
HLA-DRB11101	73.00	87.79	36.78	0.282	0.776	213	87

Table S23: Performance of LA method on MHCBN-SRDS2 datasets using 5-fold cross-validation test.

allele	ACC	Sn	Sp	CC	AUC	binders	Non-binders
HLA-DR1	68.39	82.51	38.68	0.231	0.717	223	106
HLA-DR2	62.32	71.24	51.22	0.229	0.665	153	123
HLA-DR5	47.85	15.00	68.22	-0.188	0.459	80	129
HLA-DRB10101	77.18	92.11	22.37	0.190	0.705	279	76
HLA-DRB10301	55.88	54.33	57.24	0.116	0.563	127	145
HLA-DRB10401	75.72	91.09	23.53	0.187	0.658	404	119
HLA-DRB10701	66.97	86.84	21.21	0.102	0.617	152	66
HLA-DRB11101	66.54	82.42	33.33	0.176	0.705	182	87

Table S24: Performance of LA method on MHCBN-SRDS3 datasets using 5-fold cross-validation test.

allele	ACC	Sn	Sp	CC	AUC	binders	Non-binders
HLA-DR1	74.29	84.94	53.08	0.400	0.768	259	130
HLA-DR2	70.87	78.45	59.06	0.381	0.741	232	149
HLA-DR5	58.27	35.00	73.38	0.089	0.616	100	154
HLA-DRB10101	83.47	93.85	47.32	0.476	0.819	390	112
HLA-DRB10301	67.18	61.14	72.09	0.334	0.736	175	215
HLA-DRB10401	79.78	92.45	40.23	0.387	0.760	543	174
HLA-DRB10701	80.33	91.08	55.43	0.508	0.794	213	92
HLA-DRB11101	75.14	84.94	57.25	0.440	0.816	239	131

Table S25: Performance of LA method on MHCBN-WUPDS datasets using 5-fold cross-validation test.

allele	ACC	Sn	Sp	CC	AUC
HLA-DR1	75.32	92.37	40.28	0.396	0.747
HLA-DR2	70.50	83.06	55.23	0.402	0.717
HLA-DR5	62.10	18.96	89.06	0.112	0.594
HLA-DRB10101	71.91	90.23	12.16	0.033	0.672
HLA-DRB10301	66.66	64.47	68.53	0.330	0.710
HLA-DRB10401	84.24	95.55	27.67	0.313	0.757
HLA-DRB10701	75.85	91.99	37.50	0.361	0.724
HLA-DRB11101	76.42	88.54	51.90	0.440	0.820

Table S26: Performance of SVM using 5-spectrum kernel method on MHCBN-UPDS datasets using 5-fold cross-validation test.

allele	ACC	Sn	Sp	CC	AUC	binders	Non-binders
HLA-DR1	77.94	86.32	48.33	0.351	0.747	636	180
HLA-DR2	74.66	75.48	72.62	0.448	0.806	416	168
HLA-DR5	71.61	67.89	76.30	0.439	0.743	218	173
HLA-DRB10101	83.89	90.58	55.91	0.474	0.758	531	127
HLA-DRB10301	70.26	82.76	56.09	0.405	0.770	261	230
HLA-DRB10401	76.84	82.24	55.22	0.345	0.705	805	201
HLA-DRB10701	77.69	79.11	73.83	0.491	0.778	292	107
HLA-DRB11101	66.67	62.78	76.64	0.354	0.754	352	137

Table S27: Performance of SVM using 5-spectrum kernel method on MHCBN-SRDS1 datasets using 5-fold cross-validation test.

allele	ACC	Sn	Sp	CC	AUC	binders	Non-binders
HLA-DR1	72.89	89.63	23.42	0.165	0.545	328	111
HLA-DR2	39.88	19.80	71.77	-0.097	0.456	197	124
HLA-DR5	51.65	69.37	36.64	0.063	0.533	111	131
HLA-DRB10101	69.49	83.38	18.18	0.017	0.456	325	88
HLA-DRB10301	42.16	63.50	22.67	-0.152	0.323	137	150
HLA-DRB10401	68.04	81.95	19.85	0.019	0.381	471	136
HLA-DRB10701	46.96	49.72	39.71	-0.095	0.424	179	68
HLA-DRB11101	54.33	57.75	45.98	0.034	0.493	213	87

Table S28: Performance of SVM using 5-spectrum kernel method on MHCBN-SRDS2 datasets using 5-fold cross-validation test.

allele	ACC	Sn	Sp	CC	AUC	binders	Non-binders
HLA-DR1	66.26	87.00	22.64	0.123	0.448	223	106
HLA-DR2	34.42	15.69	57.72	-0.296	0.374	153	123
HLA-DR5	43.06	52.50	37.21	-0.102	0.369	80	129
HLA-DRB10101	56.34	66.31	19.74	-0.124	0.351	279	76
HLA-DRB10301	35.29	37.01	33.79	-0.292	0.273	127	145
HLA-DRB10401	43.02	49.01	22.69	-0.239	0.261	404	119
HLA-DRB10701	62.84	78.95	25.76	0.052	0.414	152	66
HLA-DRB11101	33.46	22.53	56.32	-0.217	0.386	182	87

Table S29: Performance of SVM using 5-spectrum kernel method on MHCBN-SRDS3 datasets using 5-fold cross-validation test.

allele	ACC	Sn	Sp	CC	AUC	binders	Non-binders
HLA-DR1	73.78	86.87	47.69	0.378	0.685	259	130
HLA-DR2	62.99	56.47	73.15	0.291	0.709	232	149
HLA-DR5	58.27	30.00	76.62	0.074	0.557	100	154
HLA-DRB10101	77.29	83.08	57.14	0.382	0.691	390	112
HLA-DRB10301	63.08	49.71	73.95	0.244	0.678	175	215
HLA-DRB10401	75.59	83.06	52.30	0.348	0.624	543	174
HLA-DRB10701	62.62	58.22	72.83	0.285	0.737	213	92
HLA-DRB11101	59.19	47.28	80.92	0.279	0.755	239	131

Table S30: Performance of SVM using 5-spectrum kernel method on MHCBN-WUPDS datasets using 5-fold cross-validation test.

allele	ACC	Sn	Sp	CC	AUC
HLA-DR1	72.93	93.86	29.93	0.322	0.655
HLA-DR2	62.87	81.00	40.84	0.240	0.636
HLA-DR5	57.24	26.36	76.53	0.033	0.518
HLA-DRB10101	72.23	90.87	11.42	0.033	0.535
HLA-DRB10301	59.97	58.70	61.04	0.197	0.648
HLA-DRB10401	80.67	92.38	22.13	0.180	0.536
HLA-DRB10701	75.52	84.63	53.91	0.397	0.667
HLA-DRB11101	71.91	86.86	41.67	0.321	0.676

Table S31: Performance of CTD method on IEDB-UPDS datasets using 5-fold cross-validation test.

allele	ACC	Sn	Sp	CC	AUC	binders	Non-binders
HLA-DRB1-0101	79.12	88.78	54.40	0.459	0.770	1105	432
HLA-DRB1-0301	86.25	35.56	98.56	0.496	0.788	135	556
HLA-DRB1-0401	73.94	69.72	77.18	0.469	0.790	317	412
HLA-DRB1-0404	75.92	73.45	78.03	0.515	0.821	113	132
HLA-DRB1-0405	73.28	72.57	73.95	0.465	0.788	113	119
HLA-DRB1-0701	72.08	64.91	77.48	0.427	0.757	228	302
HLA-DRB1-0802	74.59	56.92	84.17	0.427	0.793	65	120
HLA-DRB1-1101	79.61	43.65	96.84	0.513	0.798	197	411
HLA-DRB1-1302	73.73	78.29	66.99	0.454	0.775	152	103
HLA-DRB1-1501	71.92	71.00	72.79	0.438	0.779	269	283
HLA-DRB4-0101	72.64	35.87	88.37	0.284	0.738	92	215
HLA-DRB5-0101	75.84	51.16	89.92	0.456	0.785	215	377

Table S32: Performance of CTD method on IEDB-SRDS1 datasets using 5-fold cross-validation test.

allele	ACC	Sn	Sp	CC	AUC	binders	Non-binders
HLA-DRB1-0101	71.74	96.12	13.06	0.169	0.604	645	268
HLA-DRB1-0301	81.08	28.21	95.21	0.322	0.605	78	292
HLA-DRB1-0401	64.05	54.82	70.99	0.261	0.671	197	262
HLA-DRB1-0404	69.82	57.97	78.00	0.367	0.703	69	100
HLA-DRB1-0405	61.01	58.11	63.53	0.216	0.654	74	85
HLA-DRB1-0701	58.86	42.86	70.44	0.138	0.605	147	203
HLA-DRB1-0802	68.03	43.48	79.21	0.235	0.692	46	101
HLA-DRB1-1101	68.53	39.34	84.86	0.273	0.698	122	218
HLA-DRB1-1302	61.29	72.38	46.91	0.199	0.627	105	81
HLA-DRB1-1501	59.82	50.30	68.75	0.194	0.623	165	176
HLA-DRB4-0101	67.39	7.81	99.17	0.187	0.547	64	120
HLA-DRB5-0101	64.81	48.78	74.63	0.239	0.638	123	201

Table S33: Performance of CTD method on IEDB-SRDS2 datasets using 5-fold cross-validation test.

allele	ACC	Sn	Sp	CC	AUC	binders	Non-binders
HLA-DRB1-0101	71.95	87.96	33.72	0.255	0.664	623	261
HLA-DRB1-0301	79.42	28.99	92.03	0.257	0.673	69	276
HLA-DRB1-0401	64.04	55.68	69.80	0.255	0.662	176	255
HLA-DRB1-0404	68.13	51.61	78.57	0.312	0.664	62	98
HLA-DRB1-0405	53.90	47.14	59.52	0.067	0.576	70	84
HLA-DRB1-0701	61.65	48.18	70.79	0.193	0.609	137	202
HLA-DRB1-0802	70.55	47.83	81.00	0.298	0.732	46	100
HLA-DRB1-1101	65.94	39.64	79.72	0.207	0.666	111	212
HLA-DRB1-1302	59.55	60.82	58.02	0.188	0.594	97	81
HLA-DRB1-1501	60.76	51.41	68.39	0.201	0.636	142	174
HLA-DRB4-0101	65.38	28.57	84.87	0.161	0.644	63	119
HLA-DRB5-0101	62.21	13.27	90.72	0.062	0.492	113	194

Table S34: Performance of CTD method on IEDB-SRDS3 datasets using 5-fold cross-validation test.

allele	ACC	Sn	Sp	CC	AUC	binders	Non-binders
HLA-DRB1-0101	76.29	88.91	43.84	0.366	0.745	938	365
HLA-DRB1-0301	79.66	17.28	92.42	0.126	0.669	81	396
HLA-DRB1-0401	68.83	51.16	80.00	0.325	0.730	215	340
HLA-DRB1-0404	67.21	52.70	77.06	0.306	0.683	74	109
HLA-DRB1-0405	56.47	56.79	56.18	0.130	0.598	81	89
HLA-DRB1-0701	67.79	55.49	75.55	0.314	0.714	173	274
HLA-DRB1-0802	68.15	38.78	81.48	0.217	0.696	49	108
HLA-DRB1-1101	73.23	43.17	85.98	0.318	0.699	139	328
HLA-DRB1-1302	66.83	70.91	61.96	0.330	0.686	110	92
HLA-DRB1-1501	63.37	53.51	70.38	0.241	0.671	185	260
HLA-DRB4-0101	76.49	48.24	88.50	0.403	0.759	85	200
HLA-DRB5-0101	66.67	31.97	83.17	0.172	0.644	147	309

Table S35: Performance of CTD method on IEDB-WUPDS datasets using 5-fold cross-validation test.

allele	ACC	Sn	Sp	CC	AUC
HLA-DRB1-0101	71.76	87.15	38.11	0.289	0.692
HLA-DRB1-0301	85.19	19.52	99.18	0.358	0.640
HLA-DRB1-0401	66.24	55.99	73.04	0.292	0.686
HLA-DRB1-0404	69.61	51.42	81.12	0.341	0.727
HLA-DRB1-0405	63.79	57.24	69.25	0.267	0.672
HLA-DRB1-0701	67.52	50.75	77.89	0.296	0.676
HLA-DRB1-0802	72.36	12.45	98.99	0.254	0.598
HLA-DRB1-1101	73.30	26.62	95.35	0.319	0.676
HLA-DRB1-1302	64.25	57.07	72.56	0.298	0.683
HLA-DRB1-1501	65.20	58.24	70.25	0.285	0.686
HLA-DRB4-0101	70.43	9.15	97.18	0.137	0.610
HLA-DRB5-0101	68.41	8.63	99.40	0.212	0.569

Table S36: Performance of LA method on IEDB-UPDS datasets using 5-fold cross-validation test.

allele	ACC	Sn	Sp	CC	AUC	binders	Non-binders
HLA-DRB1-0101	78.46	91.76	44.44	0.420	0.767	1105	432
HLA-DRB1-0301	81.33	29.63	93.88	0.301	0.822	135	556
HLA-DRB1-0401	70.78	61.83	77.67	0.401	0.786	317	412
HLA-DRB1-0404	71.43	64.60	77.27	0.423	0.763	113	132
HLA-DRB1-0405	70.69	65.49	75.63	0.414	0.770	113	119
HLA-DRB1-0701	72.26	61.40	80.46	0.428	0.799	228	302
HLA-DRB1-0802	78.92	52.31	93.33	0.520	0.896	65	120
HLA-DRB1-1101	74.18	46.19	87.59	0.374	0.822	197	411
HLA-DRB1-1302	76.47	84.21	65.05	0.505	0.865	152	103
HLA-DRB1-1501	71.38	66.91	75.62	0.427	0.781	269	283
HLA-DRB4-0101	78.50	50.00	90.70	0.454	0.795	92	215
HLA-DRB5-0101	69.09	38.60	86.47	0.288	0.755	215	377

Table S37: Performance of LA method on IEDB-SRDS1 datasets using 5-fold cross-validation test.

allele	ACC	Sn	Sp	CC	AUC	binders	Non-binders
HLA-DRB1-0101	71.52	85.89	36.94	0.256	0.628	645	268
HLA-DRB1-0301	78.11	28.21	91.44	0.241	0.668	78	292
HLA-DRB1-0401	60.13	49.75	67.94	0.179	0.619	197	262
HLA-DRB1-0404	66.86	47.83	80.00	0.295	0.644	69	100
HLA-DRB1-0405	59.12	48.65	68.24	0.172	0.557	74	85
HLA-DRB1-0701	62.86	51.70	70.94	0.230	0.658	147	203
HLA-DRB1-0802	73.47	32.61	92.08	0.315	0.762	46	101
HLA-DRB1-1101	63.82	31.15	82.11	0.152	0.668	122	218
HLA-DRB1-1302	66.67	79.05	50.62	0.311	0.750	105	81
HLA-DRB1-1501	56.01	50.91	60.80	0.118	0.595	165	176
HLA-DRB4-0101	64.13	39.06	77.50	0.175	0.611	64	120
HLA-DRB5-0101	60.49	35.77	75.62	0.122	0.578	123	201

Table S38: Performance of LA method on IEDB-SRDS2 datasets using 5-fold cross-validation test.

allele	ACC	Sn	Sp	CC	AUC	binders	Non-binders
HLA-DRB1-0101	70.93	86.36	34.10	0.235	0.629	623	261
HLA-DRB1-0301	76.81	13.04	92.75	0.084	0.657	69	276
HLA-DRB1-0401	60.32	45.45	70.59	0.164	0.592	176	255
HLA-DRB1-0404	61.25	40.32	74.49	0.156	0.578	62	98
HLA-DRB1-0405	55.84	45.71	64.29	0.102	0.523	70	84
HLA-DRB1-0701	61.06	44.53	72.28	0.173	0.611	137	202
HLA-DRB1-0802	69.86	23.91	91.00	0.201	0.732	46	100
HLA-DRB1-1101	62.54	32.43	78.30	0.117	0.602	111	212
HLA-DRB1-1302	62.36	68.04	55.56	0.238	0.681	97	81
HLA-DRB1-1501	53.48	46.48	59.20	0.057	0.534	142	174
HLA-DRB4-0101	63.19	30.16	80.67	0.122	0.627	63	119
HLA-DRB5-0101	54.72	29.20	69.59	-0.013	0.503	113	194

Table S39: Performance of LA method on IEDB-SRDS3 datasets using 5-fold cross-validation test.

allele	ACC	Sn	Sp	CC	AUC	binders	Non-binders
HLA-DRB1-0101	77.36	89.45	46.30	0.397	0.754	938	365
HLA-DRB1-0301	81.55	18.52	94.44	0.182	0.747	81	396
HLA-DRB1-0401	68.83	54.42	77.94	0.331	0.736	215	340
HLA-DRB1-0404	63.93	47.30	75.23	0.234	0.655	74	109
HLA-DRB1-0405	60.00	55.56	64.04	0.197	0.589	81	89
HLA-DRB1-0701	69.13	54.34	78.47	0.337	0.731	173	274
HLA-DRB1-0802	68.15	16.33	91.67	0.119	0.732	49	108
HLA-DRB1-1101	72.81	35.97	88.41	0.285	0.754	139	328
HLA-DRB1-1302	68.32	74.55	60.87	0.358	0.767	110	92
HLA-DRB1-1501	64.94	54.59	72.31	0.272	0.712	185	260
HLA-DRB4-0101	76.84	50.59	88.00	0.416	0.785	85	200
HLA-DRB5-0101	68.64	34.01	85.11	0.219	0.679	147	309

Table S40: Performance of LA method on IEDB-WUPDS datasets using 5-fold cross-validation test.

allele	ACC	Sn	Sp	CC	AUC
HLA-DRB1-0101	74.79	89.77	42.02	0.367	0.703
HLA-DRB1-0301	82.16	24.74	94.39	0.255	0.742
HLA-DRB1-0401	64.52	48.51	75.16	0.244	0.697
HLA-DRB1-0404	66.04	51.89	75.00	0.274	0.668
HLA-DRB1-0405	64.18	56.43	70.63	0.273	0.680
HLA-DRB1-0701	67.85	48.44	79.85	0.297	0.728
HLA-DRB1-0802	75.35	36.93	92.42	0.364	0.849
HLA-DRB1-1101	69.33	38.89	83.71	0.249	0.742
HLA-DRB1-1302	67.80	76.32	57.93	0.349	0.778
HLA-DRB1-1501	64.88	54.00	72.78	0.272	0.681
HLA-DRB4-0101	73.53	41.13	87.68	0.325	0.724
HLA-DRB5-0101	65.84	29.42	84.72	0.167	0.665

Table S41: Performance of SVM using 5-spectrum kernel method on IEDB-UPDS datasets using 5-fold cross-validation test.

allele	ACC	Sn	Sp	CC	AUC	binders	Non-binders
HLA-DRB1-0101	80.03	92.94	46.99	0.464	0.806	1105	432
HLA-DRB1-0301	80.32	59.26	85.43	0.419	0.771	135	556
HLA-DRB1-0401	71.60	50.79	87.62	0.419	0.769	317	412
HLA-DRB1-0404	66.94	51.33	80.30	0.332	0.639	113	132
HLA-DRB1-0405	68.10	47.79	87.39	0.385	0.693	113	119
HLA-DRB1-0701	69.62	50.00	84.44	0.371	0.727	228	302
HLA-DRB1-0802	74.05	38.46	93.33	0.396	0.703	65	120
HLA-DRB1-1101	76.48	52.28	88.08	0.436	0.800	197	411
HLA-DRB1-1302	67.84	52.63	90.29	0.441	0.783	152	103
HLA-DRB1-1501	71.74	63.20	79.86	0.437	0.794	269	283
HLA-DRB4-0101	59.93	81.52	50.70	0.300	0.703	92	215
HLA-DRB5-0101	73.99	53.95	85.41	0.418	0.784	215	377

Table S42: Performance of SVM using 5-spectrum kernel method on IEDB-SRDS1 datasets using 5-fold cross-validation test.

allele	ACC	Sn	Sp	CC	AUC	binders	Non-binders
HLA-DRB1-0101	57.28	71.63	22.76	-0.058	0.427	645	268
HLA-DRB1-0301	75.14	24.36	88.70	0.153	0.460	78	292
HLA-DRB1-0401	52.07	15.23	79.77	-0.064	0.444	197	262
HLA-DRB1-0404	46.15	42.03	49.00	-0.088	0.411	69	100
HLA-DRB1-0405	45.28	4.05	81.18	-0.227	0.331	74	85
HLA-DRB1-0701	52.00	31.97	66.50	-0.016	0.445	147	203
HLA-DRB1-0802	62.59	4.35	89.11	-0.107	0.378	46	101
HLA-DRB1-1101	59.12	13.11	84.86	-0.028	0.454	122	218
HLA-DRB1-1302	50.00	22.86	85.19	0.101	0.549	105	81
HLA-DRB1-1501	49.85	38.18	60.80	-0.010	0.460	165	176
HLA-DRB4-0101	57.61	10.94	82.50	-0.087	0.444	64	120
HLA-DRB5-0101	50.31	8.94	75.62	-0.193	0.344	123	201

Table S43: Performance of SVM using 5-spectrum kernel method on IEDB-SRDS2 datasets using 5-fold cross-validation test.

allele	ACC	Sn	Sp	CC	AUC	binders	Non-binders
HLA-DRB1-0101	52.49	60.67	32.95	-0.060	0.436	623	261
HLA-DRB1-0301	64.64	26.09	74.28	0.003	0.401	69	276
HLA-DRB1-0401	45.48	34.09	53.33	-0.125	0.371	176	255
HLA-DRB1-0404	41.25	45.16	38.78	-0.157	0.364	62	98
HLA-DRB1-0405	47.40	5.71	82.14	-0.184	0.340	70	84
HLA-DRB1-0701	43.95	51.09	39.11	-0.097	0.389	137	202
HLA-DRB1-0802	63.01	4.35	90.00	-0.096	0.370	46	100
HLA-DRB1-1101	57.89	4.50	85.85	-0.147	0.399	111	212
HLA-DRB1-1302	47.75	25.77	74.07	-0.002	0.483	97	81
HLA-DRB1-1501	44.94	36.62	51.72	-0.117	0.380	142	174
HLA-DRB4-0101	59.89	11.11	85.71	-0.045	0.466	63	119
HLA-DRB5-0101	47.23	2.65	73.20	-0.304	0.278	113	194

Table S44: Performance of SVM using 5-spectrum kernel method on IEDB-SRDS3 datasets using 5-fold cross-validation test.

allele	ACC	Sn	Sp	CC	AUC	binders	Non-binders
HLA-DRB1-0101	76.44	91.90	36.71	0.349	0.742	938	365
HLA-DRB1-0301	73.58	29.63	82.58	0.116	0.590	81	396
HLA-DRB1-0401	59.46	39.07	72.35	0.119	0.593	215	340
HLA-DRB1-0404	48.09	56.76	42.20	-0.010	0.544	74	109
HLA-DRB1-0405	51.76	18.52	82.02	0.007	0.414	81	89
HLA-DRB1-0701	61.30	37.57	76.28	0.149	0.628	173	274
HLA-DRB1-0802	67.52	14.29	91.67	0.091	0.515	49	108
HLA-DRB1-1101	63.38	59.71	64.94	0.228	0.673	139	328
HLA-DRB1-1302	57.43	30.91	89.13	0.242	0.669	110	92
HLA-DRB1-1501	62.92	76.22	53.46	0.297	0.681	185	260
HLA-DRB4-0101	61.75	68.24	59.00	0.249	0.699	85	200
HLA-DRB5-0101	64.25	27.89	81.55	0.107	0.591	147	309

Table S45: Performance of SVM using 5-spectrum kernel method on IEDB-WUPDS datasets using 5-fold cross-validation test.

allele	ACC	Sn	Sp	CC	AUC
HLA-DRB1-0101	72.61	93.49	26.94	0.283	0.685
HLA-DRB1-0301	82.67	27.54	94.41	0.285	0.715
HLA-DRB1-0401	55.54	45.29	62.35	0.076	0.601
HLA-DRB1-0404	55.42	52.69	57.14	0.096	0.551
HLA-DRB1-0405	59.46	36.29	78.77	0.167	0.604
HLA-DRB1-0701	59.19	53.00	63.01	0.157	0.609
HLA-DRB1-0802	49.86	65.45	42.93	0.079	0.582
HLA-DRB1-1101	70.49	30.67	89.30	0.247	0.666
HLA-DRB1-1302	56.06	66.73	43.70	0.107	0.586
HLA-DRB1-1501	63.34	44.78	76.82	0.228	0.670
HLA-DRB4-0101	67.20	52.95	73.42	0.255	0.665
HLA-DRB5-0101	62.21	29.56	79.14	0.097	0.618

Table S46: AUC values for the three prediction methods evaluated on IEDB-UPDS datasets. For each dataset, the rank of each classifier is shown in parentheses.

allele	5-spectrum	LA	CTD
HLA-DRB1-0101	0.806(1)	0.767(3)	0.770(2)
HLA-DRB1-0301	0.771(3)	0.822(1)	0.788(2)
HLA-DRB1-0401	0.769(3)	0.786(2)	0.790(1)
HLA-DRB1-0404	0.639(3)	0.763(2)	0.821(1)
HLA-DRB1-0405	0.693(3)	0.770(2)	0.788(1)
HLA-DRB1-0701	0.727(3)	0.799(1)	0.757(2)
HLA-DRB1-0802	0.703(3)	0.896(1)	0.793(2)
HLA-DRB1-1101	0.800(2)	0.822(1)	0.798(3)
HLA-DRB1-1302	0.783(2)	0.865(1)	0.775(3)
HLA-DRB1-1501	0.794(1)	0.781(2)	0.779(3)
HLA-DRB4-0101	0.703(3)	0.795(1)	0.738(2)
HLA-DRB5-0101	0.784(2)	0.755(3)	0.785(1)
Average	0.748(2.42)	0.802(1.67)	0.782(1.92)

Table S47: AUC values for the three prediction methods evaluated on IEDB-SRDS1 datasets. For each dataset, the rank of each classifier is shown in parentheses.

allele	5-spectrum	LA	CTD
HLA-DRB1-0101	0.427(3)	0.628(1)	0.604(2)
HLA-DRB1-0301	0.460(3)	0.668(1)	0.605(2)
HLA-DRB1-0401	0.444(3)	0.619(2)	0.671(1)
HLA-DRB1-0404	0.411(3)	0.644(2)	0.703(1)
HLA-DRB1-0405	0.331(3)	0.557(2)	0.654(1)
HLA-DRB1-0701	0.445(3)	0.658(1)	0.605(2)
HLA-DRB1-0802	0.378(3)	0.762(1)	0.692(2)
HLA-DRB1-1101	0.454(3)	0.668(2)	0.698(1)
HLA-DRB1-1302	0.549(3)	0.750(1)	0.627(2)
HLA-DRB1-1501	0.460(3)	0.595(2)	0.623(1)
HLA-DRB4-0101	0.444(3)	0.611(1)	0.547(2)
HLA-DRB5-0101	0.344(3)	0.578(2)	0.638(1)
Average	0.429(3)	0.645(1.5)	0.639(1.5)

Table S48: AUC values for the three prediction methods evaluated on IEDB-SRDS2 datasets. For each dataset, the rank of each classifier is shown in parentheses.

allele	5-spectrum	LA	CTD
HLA-DRB1-0101	0.436(3)	0.629(2)	0.664(1)
HLA-DRB1-0301	0.401(3)	0.657(2)	0.673(1)
HLA-DRB1-0401	0.371(3)	0.592(2)	0.662(1)
HLA-DRB1-0404	0.364(3)	0.578(2)	0.664(1)
HLA-DRB1-0405	0.340(3)	0.523(2)	0.576(1)
HLA-DRB1-0701	0.389(3)	0.611(1)	0.609(2)
HLA-DRB1-0802	0.370(3)	0.732(1.5)	0.732(1.5)
HLA-DRB1-1101	0.399(3)	0.602(2)	0.666(1)
HLA-DRB1-1302	0.483(3)	0.681(1)	0.594(2)
HLA-DRB1-1501	0.380(3)	0.534(2)	0.636(1)
HLA-DRB4-0101	0.466(3)	0.627(2)	0.644(1)
HLA-DRB5-0101	0.278(3)	0.503(1)	0.492(2)
Average	0.390(3)	0.606(1.71)	0.634(1.29)

Table S49: AUC values for the three prediction methods evaluated on IEDB-SRDS3 datasets. For each dataset, the rank of each classifier is shown in parentheses.

allele	5-spectrum	LA	CTD
HLA-DRB1-0101	0.742(3)	0.754(1)	0.745(2)
HLA-DRB1-0301	0.590(3)	0.747(1)	0.669(2)
HLA-DRB1-0401	0.593(2)	0.736(1)	0.730(2)
HLA-DRB1-0404	0.544(3)	0.655(2)	0.683(1)
HLA-DRB1-0405	0.414(3)	0.589(2)	0.598(1)
HLA-DRB1-0701	0.628(3)	0.731(1)	0.714(2)
HLA-DRB1-0802	0.515(3)	0.732(1)	0.696(2)
HLA-DRB1-1101	0.673(3)	0.754(1)	0.699(2)
HLA-DRB1-1302	0.669(3)	0.767(1)	0.686(2)
HLA-DRB1-1501	0.681(2)	0.712(1)	0.671(3)
HLA-DRB4-0101	0.699(3)	0.785(1)	0.759(2)
HLA-DRB5-0101	0.591(3)	0.679(1)	0.644(2)
Average	0.612(2.83)	0.720(1.17)	0.691(1.92)

Table S50: AUC values for the three prediction methods evaluated on IEDB-WUPDS datasets. For each dataset, the rank of each classifier is shown in parentheses.

allele	5-spectrum	LA	CTD
HLA-DRB1-0101	0.685(3)	0.703(1)	0.692(2)
HLA-DRB1-0301	0.715(2)	0.742(1)	0.640(3)
HLA-DRB1-0401	0.601(3)	0.697(1)	0.686(2)
HLA-DRB1-0404	0.551(3)	0.668(2)	0.727(1)
HLA-DRB1-0405	0.604(3)	0.680(1)	0.672(2)
HLA-DRB1-0701	0.609(3)	0.728(1)	0.676(2)
HLA-DRB1-0802	0.582(3)	0.849(1)	0.598(2)
HLA-DRB1-1101	0.666(3)	0.742(1)	0.676(2)
HLA-DRB1-1302	0.586(3)	0.778(1)	0.683(2)
HLA-DRB1-1501	0.670(3)	0.681(2)	0.686(1)
HLA-DRB4-0101	0.665(2)	0.724(1)	0.610(3)
HLA-DRB5-0101	0.618(2)	0.665(1)	0.569(3)
Average	0.629(2.75)	0.721(1.17)	0.660(2.08)
